# Supplementary figures and images for: Development of landscape conservation value map of Jeju island, Korea for integrative landscape management and planning using conservation value of landscape typology
Source: PeerJ. 2021 Jun 1;9:e11449. doi: 10.7717/peerj.11449 (PMC8176906; doi:10.7717/peerj.11449)

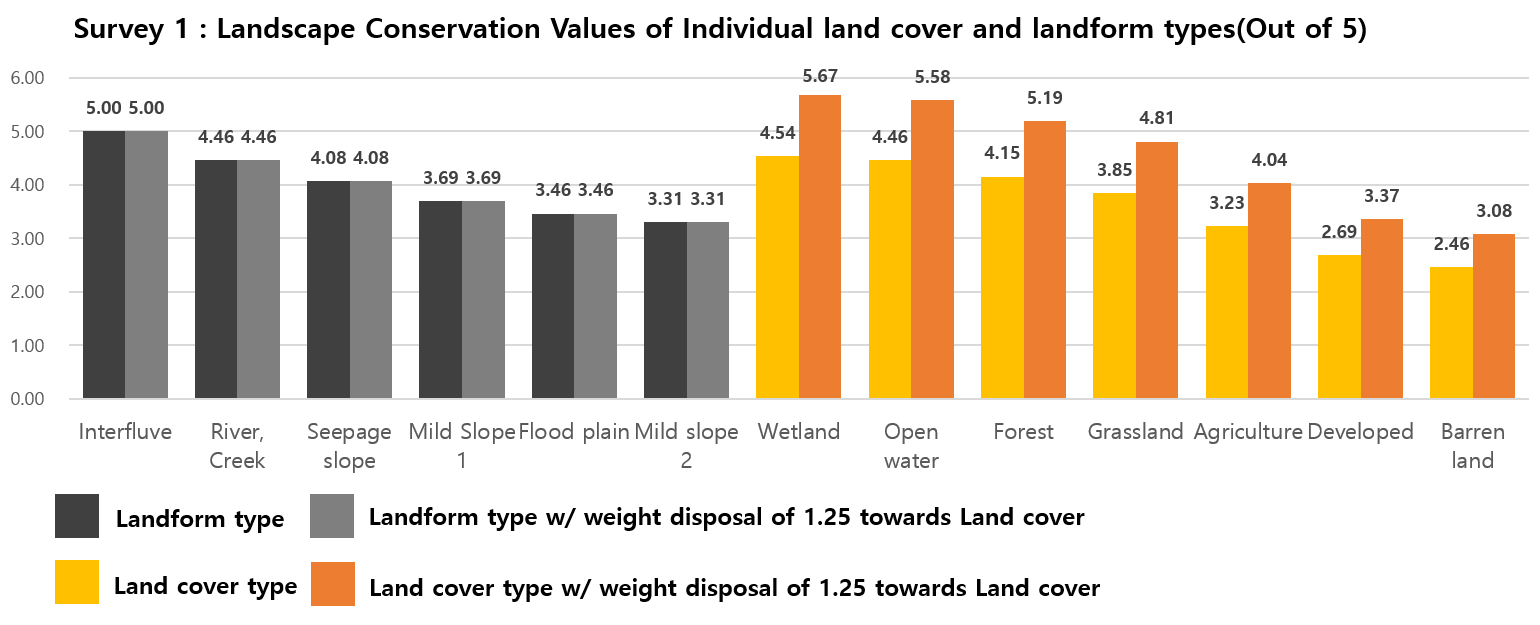

Supplement: Supplemental Information 1 — Values given to each type of variables based on the first survey with and without weight disposal of 1.25 towards land cover. On a scale of 1 to 5, the landscape conservation values for each land cover type were, in descending order, 4.53 for wetland, 4.46 for open water, 4.15 for forest, 3.84 for grassland, 3.23 for agriculture, 2.69 for developed, and 2.46 for barren land. The values for each landform type were, in descending order, 5 for summit, 4.46 for river/stream, 4.08 for shoulder, 3.69 for mild slope 1, 3.46 for flood plain, and 3.31 for mild slope 2. Landscape experts rated land cover as “somewhat important” (×1.25 weight) compared to landform; after applying a ×1.25 weight to land cover type, the mean score for land cover was 4.53 and the mean score for landform was 4.00 [file peerj-09-11449-s001.png]
