# Supplementary material for: Development of landscape conservation value map of Jeju island, Korea for integrative landscape management and planning using conservation value of landscape typology
Source: PeerJ. 2021 Jun 1;9:e11449. doi: 10.7717/peerj.11449 (PMC8176906; doi:10.7717/peerj.11449)
Supplement: Supplemental Information 2 [file peerj-09-11449-s002.docx]

**Supplemental Table S1.** Result of 2^nd^ Survey

| **Landscape Type** | **N** | **Minimum LCV** | **Maximum**  **LCV** | **Mean**  **LCV** | **Normalized**  **LCV(1 to 10)** | **Std. Deviation** |
| --- | --- | --- | --- | --- | --- | --- |
| Summit & Wetland | 13 | 8 | 10 | 9.62 | 10.00 | 0.77 |
| Summit & Open water | 13 | 9 | 10 | 9.62 | 10.00 | 0.51 |
| Summit & Forest | 13 | 6 | 10 | 9.08 | 9.41 | 1.32 |
| Summit & Grassland | 13 | 6 | 9 | 7.77 | 7.98 | 1.01 |
| Summit & Agriculture | 13 | 4 | 8 | 5.77 | 5.79 | 1.24 |
| Summit & Barren Land | 13 | 2 | 6 | 3.62 | 3.45 | 1.26 |
| Summit & Developed | 13 | 2 | 8 | 3.23 | 3.02 | 1.64 |
| Channel & Wetland | 13 | 6 | 10 | 8.31 | 8.57 | 1.25 |
| Channel & Open water | 13 | 6 | 10 | 8.15 | 8.39 | 1.14 |
| Channel & Forest | 13 | 6 | 10 | 8 | 8.23 | 1.08 |
| Channel& Grassland | 13 | 5 | 8 | 6.31 | 6.38 | 0.95 |
| Channel & Agriculture | 13 | 3 | 6 | 4.62 | 4.54 | 0.96 |
| Channel & Barren Land | 13 | 1 | 5 | 2.69 | 2.43 | 1.38 |
| Channel & Developed | 13 | 1 | 6 | 2.77 | 2.52 | 1.48 |
| Shoulder & Wetland | 13 | 5 | 10 | 7.54 | 7.73 | 1.39 |
| Shoulder & Open water | 13 | 5 | 10 | 7.92 | 8.14 | 1.5 |
| Shoulder & **Forest** | 13 | 3 | 9 | 7.46 | 7.64 | 1.66 |
| Shoulder & Grassland | 13 | 3 | 9 | 5.92 | 5.96 | 1.71 |
| Shoulder & Agriculture | 13 | 1 | 9 | 4.62 | 4.54 | 2.06 |
| Shoulder & Barren Land | 13 | 1 | 6 | 2.46 | 2.18 | 1.39 |
| Shoulder & Developed | 13 | 1 | 6 | 2.23 | 1.93 | 1.24 |
| Slope & Wetland | 13 | 5 | 9 | 6.38 | 6.46 | 1.33 |
| Slope & Open water | 13 | 4 | 9 | 6.54 | 6.64 | 1.45 |
| **Slope** & **Forest** | 13 | 3 | 8 | 5.92 | 5.96 | 1.5 |
| Slope & Grassland | 13 | 3 | 8 | 4.85 | 4.79 | 1.86 |
| **Slope** & **Agriculture** | 13 | 1 | 10 | 3.46 | 3.27 | 2.33 |
| **Slope** & **Barren Land** | 13 | 1 | 4 | 2 | 1.68 | 1.08 |
| Slope & Developed | 13 | 1 | 9 | 1.92 | 1.59 | 2.18 |
| Flat land & Wetland | 13 | 4 | 9 | 6.15 | 6.21 | 1.21 |
| Flat land & Open water | 13 | 4 | 8 | 5.85 | 5.88 | 0.99 |
| **Flat land** & **Forest** | 13 | 4 | 7 | 5.62 | 5.63 | 0.87 |
| **Flat land** & **Grassland** | 13 | 3 | 7 | 4.31 | 4.20 | 1.44 |
| **Flat land** & **Agriculture** | 13 | 1 | 5 | 3.08 | 2.86 | 1.44 |
| **Flat land** & **Barren Land** | 13 | 1 | 5 | 1.77 | 1.43 | 1.36 |
| **Flat land** & **Developed** | 13 | 1 | 3 | 1.38 | 1.00 | 0.65 |
| **Valid N (listwise)** | **13** |  |  |  |  |  |
