# Supplementary material for: Development of landscape conservation value map of Jeju island, Korea for integrative landscape management and planning using conservation value of landscape typology
Source: PeerJ. 2021 Jun 1;9:e11449. doi: 10.7717/peerj.11449 (PMC8176906; doi:10.7717/peerj.11449)
Supplement: Supplemental Information 3 — 1 Bolded landscape types represent those consisting over 2% of the whole research area [file peerj-09-11449-s003.docx]

Supplemental Table S2. Proportion of Landscape types within a research site^1^

| **Landscape Type** | **Count**  **(30m cell)** | **sq. meter** | **ha** | **Proportion (%)** |
| --- | --- | --- | --- | --- |
| Summit & Wetland | 57 | 51,300 | 5.13 | 0.00 |
| Summit & Open water | 12 | 10,800 | 1.08 | 0.00 |
| Summit & Forest | 31,864 | 28,677,600 | 2,867.76 | 1.55 |
| Summit & Grassland | 1,062 | 955,800 | 95.58 | 0.05 |
| Summit & Agriculture | 5,228 | 4,705,200 | 470.52 | 0.25 |
| Summit & Barren Land | 9,988 | 8,989,200 | 898.92 | 0.49 |
| Summit & Developed | 897 | 807,300 | 80.73 | 0.04 |
| Channel & Wetland | 2,202 | 1,981,800 | 198.18 | 0.11 |
| Channel & Open water | 516 | 464,400 | 46.44 | 0.03 |
| Channel & Forest | 9,609 | 8,648,100 | 864.81 | 0.47 |
| Channel & Grassland | 2,827 | 2,544,300 | 254.43 | 0.14 |
| Channel & Agriculture | 11,866 | 10,679,400 | 1,067.94 | 0.58 |
| Channel & Barren Land | 4,302 | 3,871,800 | 387.18 | 0.21 |
| Channel & Developed | 3,454 | 3,108,600 | 310.86 | 0.17 |
| Shoulder & Wetland | 168 | 151,200 | 15.12 | 0.01 |
| Shoulder& Open water | 81 | 72,900 | 7.29 | 0.00 |
| Shoulder & **Forest** | **113,959** | **102,563,100** | **10,256.3** | **5.55** |
| Shoulder & Grassland | 2,632 | 2,368,800 | 236.88 | 0.13 |
| Shoulder & Agriculture | 18,232 | 16,408,800 | 1,640.88 | 0.89 |
| Shoulder & Barren Land | 24,808 | 22,327,200 | 2,232.72 | 1.21 |
| Shoulder & Developed | 3,631 | 3,267,900 | 326.79 | 0.18 |
| Slope & Wetland | 626 | 563,400 | 56.34 | 0.03 |
| Slope & Open water | 290 | 261,000 | 26.1 | 0.01 |
| **Slope** & **Forest** | **221,965** | **199,768,500** | **19,976.9** | **10.81** |
| Slope & Grassland | 6,411 | 5,769,900 | 576.99 | 0.31 |
| **Slope** & **Agriculture** | **46,298** | **41,668,200** | **4,166.82** | **2.26** |
| **Slope** & **Barren Land** | **53,747** | **48,372,300** | **4,837.23** | **2.62** |
| Slope & Developed | 8,222 | 7,399,800 | 739.98 | 0.40 |
| Flat land & Wetland | 4,979 | 4,481,100 | 448.11 | 0.24 |
| Flat land & Open water | 4,574 | 4,116,600 | 411.66 | 0.22 |
| **Flat land** & **Forest** | **335,277** | **301,749,300** | **3,0174.9** | **16.33** |
| **Flat land** & **Grassland** | **46,898** | **42,208,200** | **4,220.82** | **2.28** |
| **Flat land** & **Agriculture** | **646,885** | **582,196,500** | **58,219.7** | **31.52** |
| **Flat land** & **Barren Land** | **296,850** | **267,165,000** | **26,716.5** | **14.46** |
| **Flat land** & **Developed** | **132,193** | **118,973,700** | **11,897.4** | **6.44** |
| **Total** | **2,052,610** | **1,847,349,000** | **184,735** | **100.00** |

^1^ Bolded landscape types represent those consisting over 2% of the whole research area
