# Supplementary material for: Development of landscape conservation value map of Jeju island, Korea for integrative landscape management and planning using conservation value of landscape typology
Source: PeerJ. 2021 Jun 1;9:e11449. doi: 10.7717/peerj.11449 (PMC8176906; doi:10.7717/peerj.11449)
Supplement: Supplemental Information 4 [file peerj-09-11449-s004.docx]

**Supplemental Table S3.** Images of Landscape Type

| **Landscape Type** | **Summit** |
| --- | --- |
| Wetland | 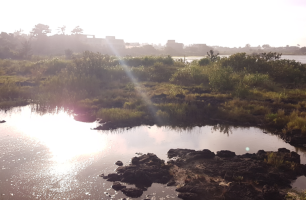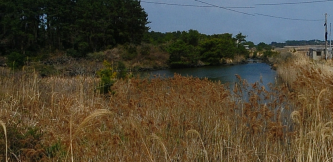 |
| Open water | 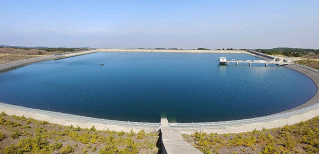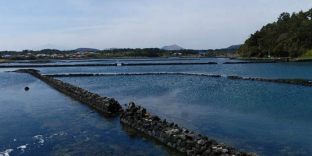 |
| Forest | 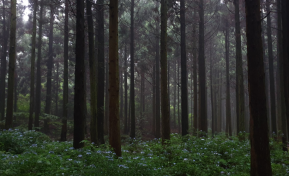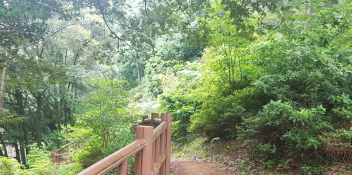 |
| Grassland | 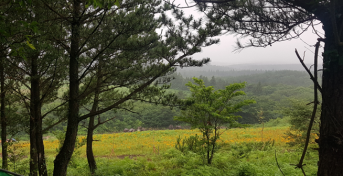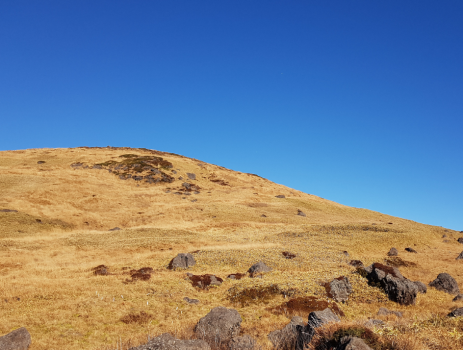 |
| Agriculture | 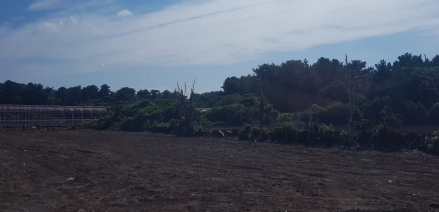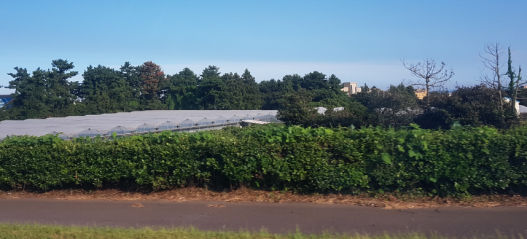 |
| Developed | 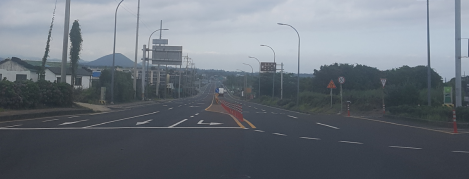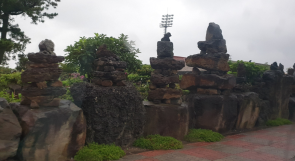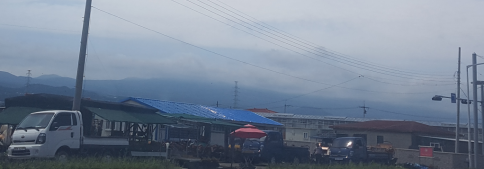 |
| Barren land | 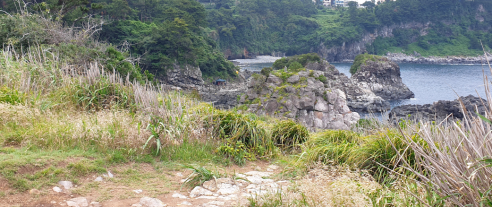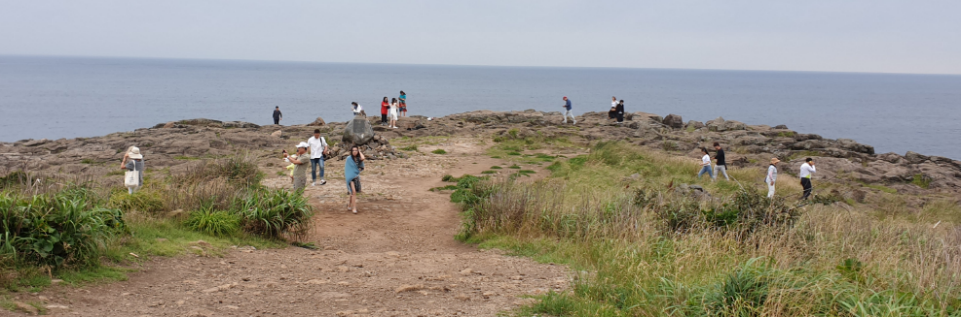 |

**Supplemental Table S3.** Images of Landscape Type(Continued)

| **Landscape type** | **Shoulder** |
| --- | --- |
| Wetland | 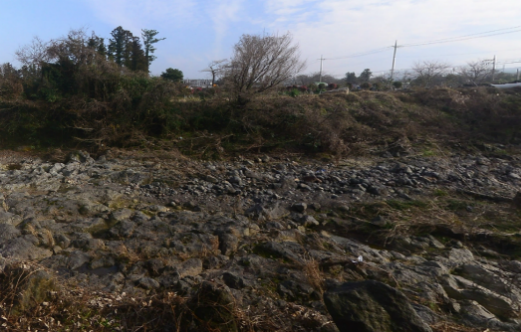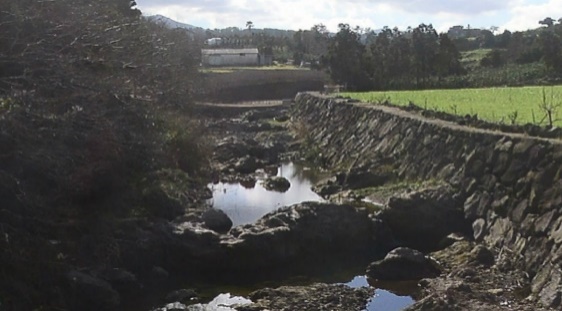 |
| Open water | 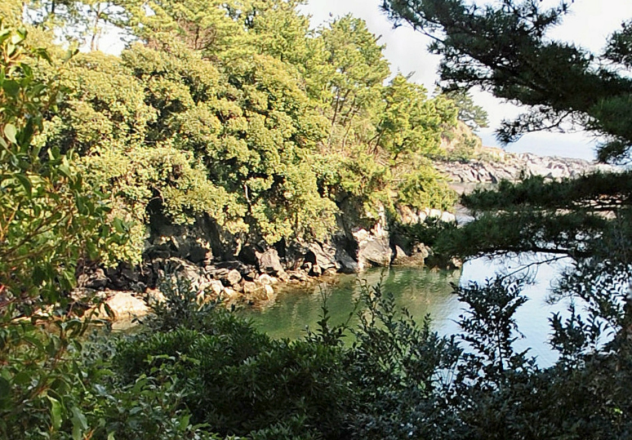 |
| Forest | 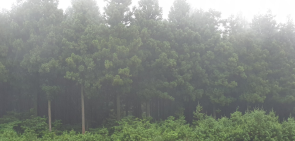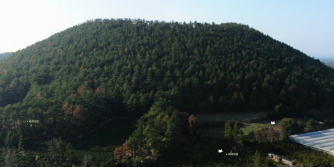 |
| Grassland | 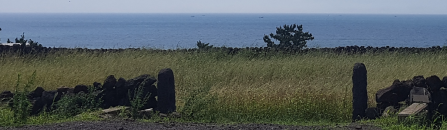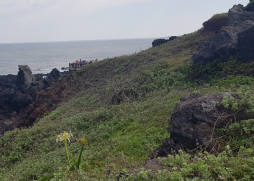 |
| Agriculture | 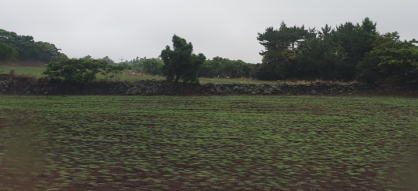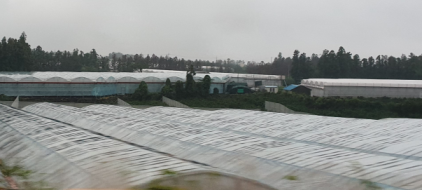 |
| Developed | 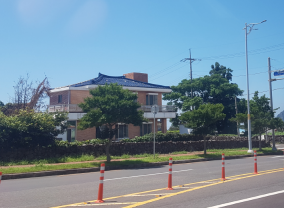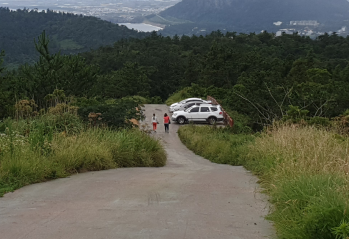 |
| Barren land | 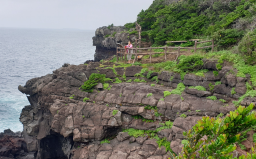 |

**Supplemental Table S3.** Images of Landscape Type(Continued)

| **Landscape type** | **Slope** |
| --- | --- |
| Wetland |  |
| Open water | 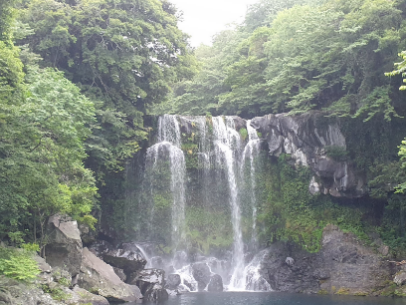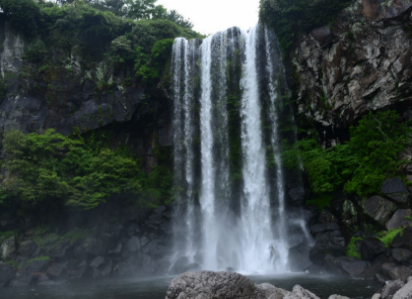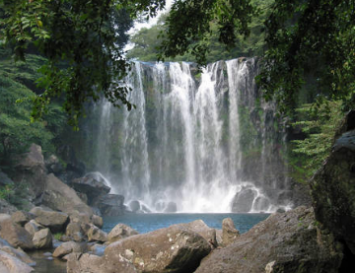 |
| Forest | 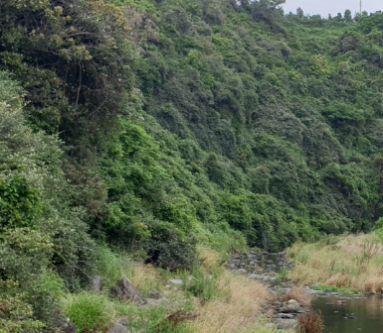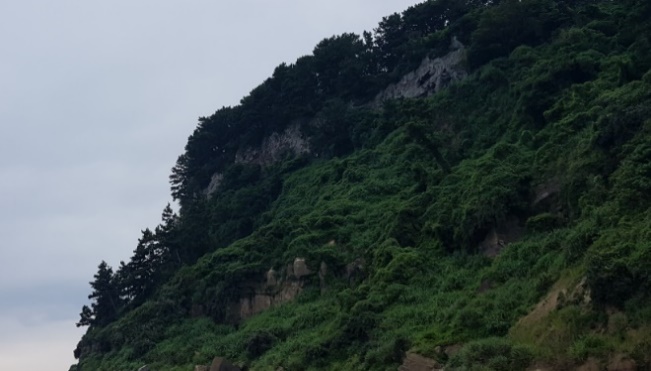 |
| Grassland | 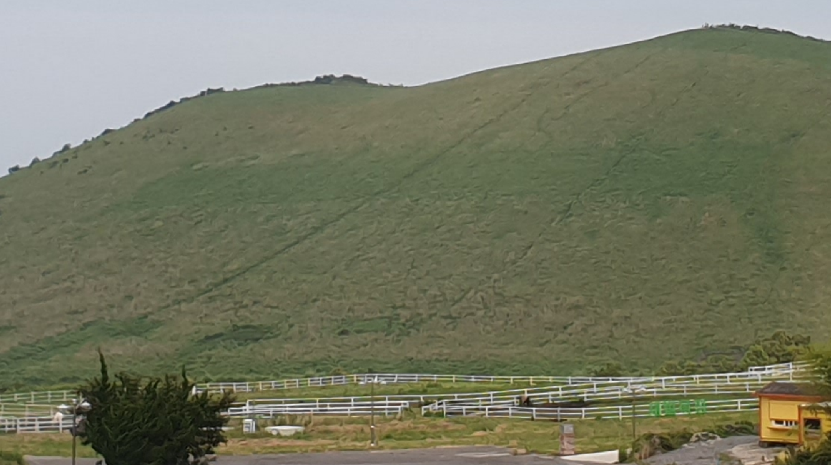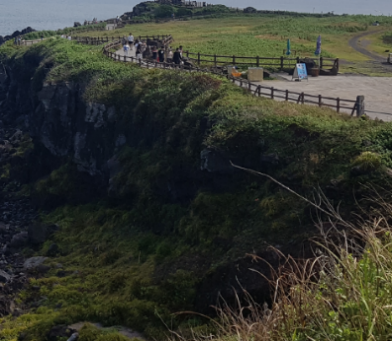 |
| Agriculture |  |
| Developed | 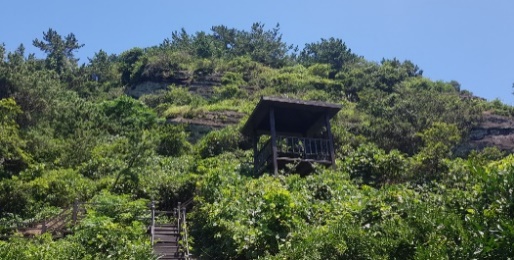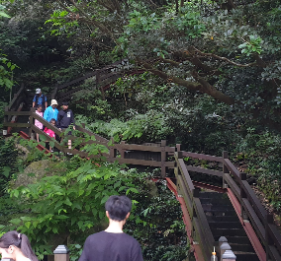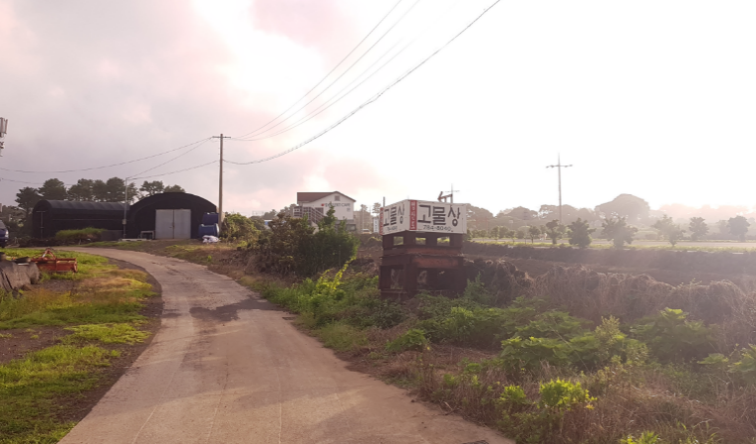 |
| Barren land | 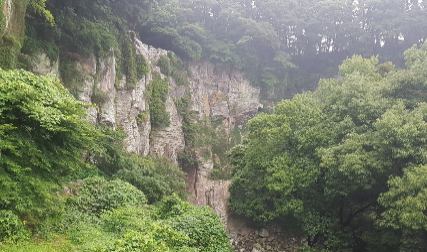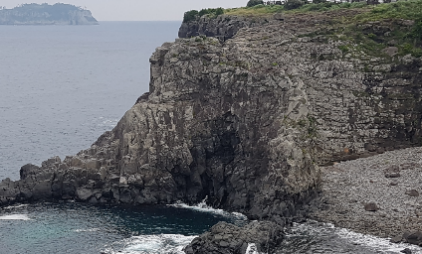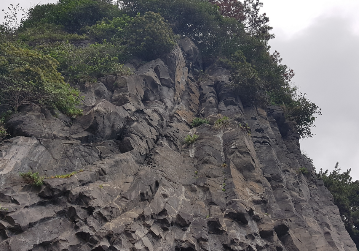 |

**Supplemental Table S3.** Images of Landscape Type(Continued)

| **Landscape type** | **Flat land** |
| --- | --- |
| Wetland | 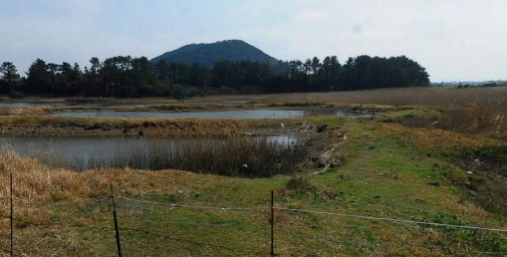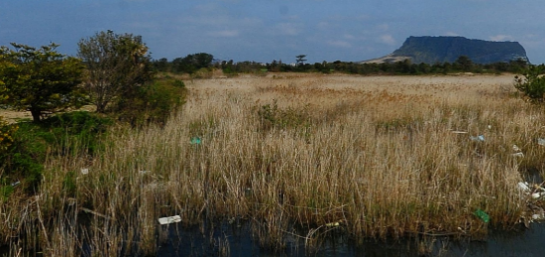 |
| Open water | 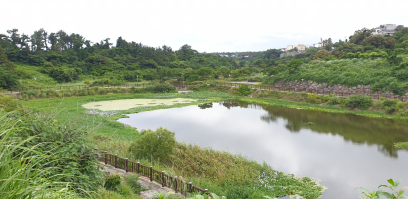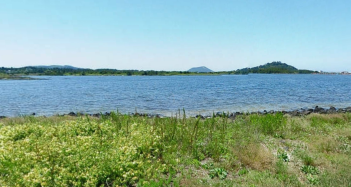 |
| Forest | 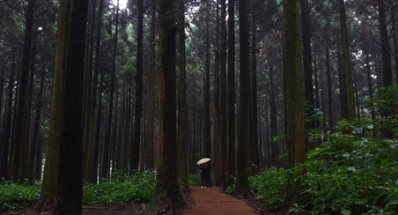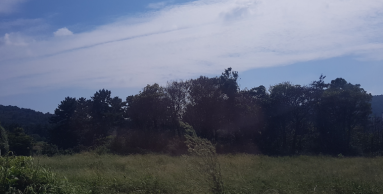 |
| Grassland | 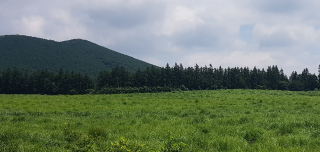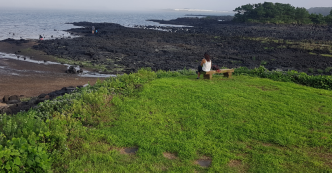 |
| Agriculture | 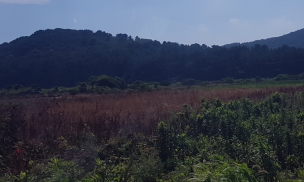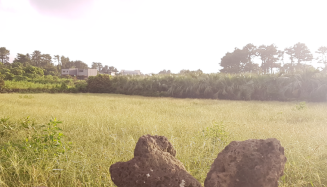 |
| Developed | 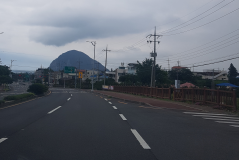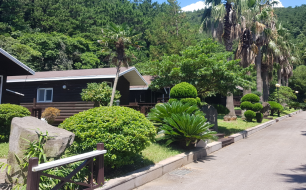 |
| Barren land | 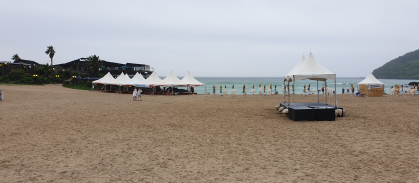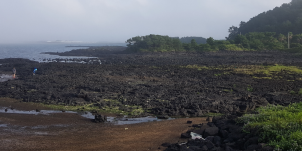 |

| **Landscape type** | **Channel** |
| --- | --- |
| Wetland | 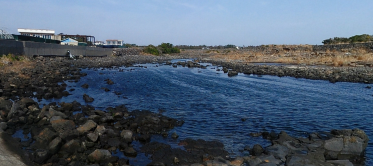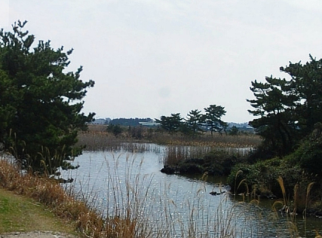 |
| Open water | 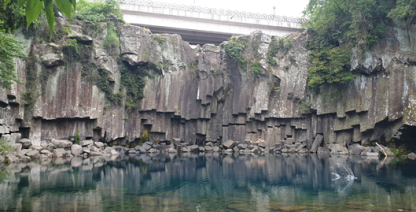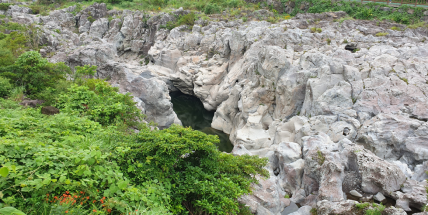 |
| Forest | 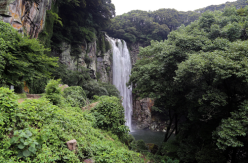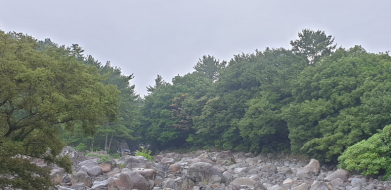 |
| Grassland | 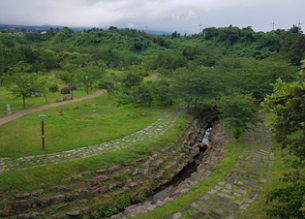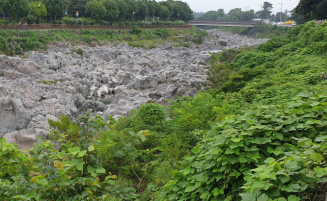 |
| Agriculture | 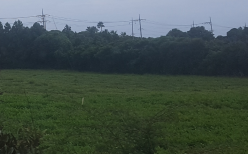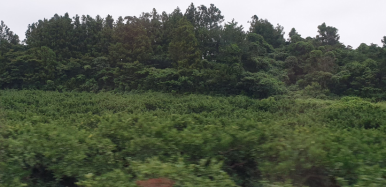 |
| Developed | 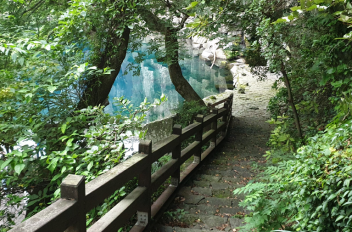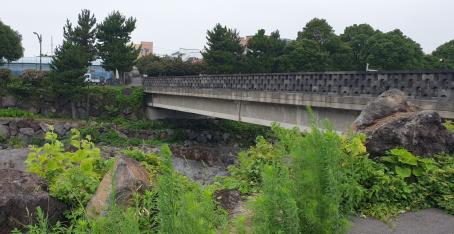 |
| Barren land | 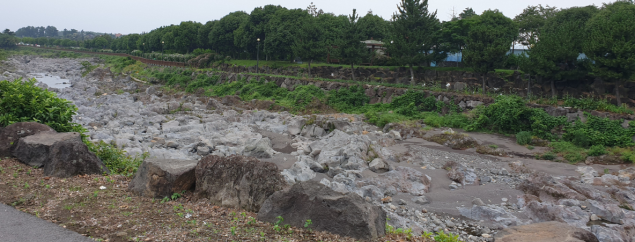 |

**Supplemental Table S3.** Images of Landscape Type(Continued)
