# Supplementary material for: Development of landscape conservation value map of Jeju island, Korea for integrative landscape management and planning using conservation value of landscape typology
Source: PeerJ. 2021 Jun 1;9:e11449. doi: 10.7717/peerj.11449 (PMC8176906; doi:10.7717/peerj.11449)
Supplement: Supplemental Information 5 — Designed through 3 rounds of FGIs [file peerj-09-11449-s005.pdf]

1st Expert Survey on Landscape Conservation Value

1st Survey

Prior to evaluating landscape conservation value of the combination of landform and land cover, We would like to ask you to give an appropriate value to each type of landform and land cover based on 6 factors(Representativeness, specificity, diversity, rarity, irreproducibility and educational value) with your expert knowledge and experience. The values are to be given separately in order to prevent bias and confusion when conducting 2nd Survey(Rating combinations of landform and land cover types).

■ Landform

1 Summit

Summit	Shoulder	Slope	Mild Slope	Flat land	Channel

Landscape Conservation Value of Summit

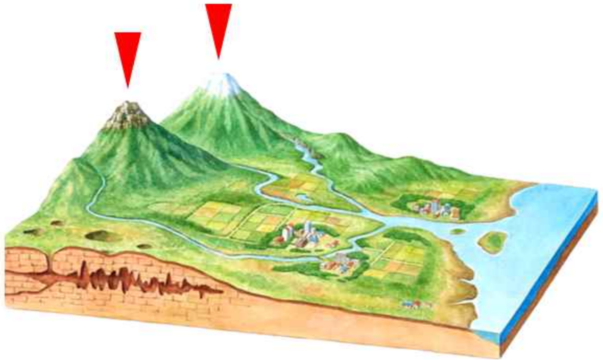

Flat land appearing at the top of the mountain limits the inflow and outflow of energy and materials from the surrounding area.

Very Low	Low	Normal	High	Very High
①	②	③	④	⑤

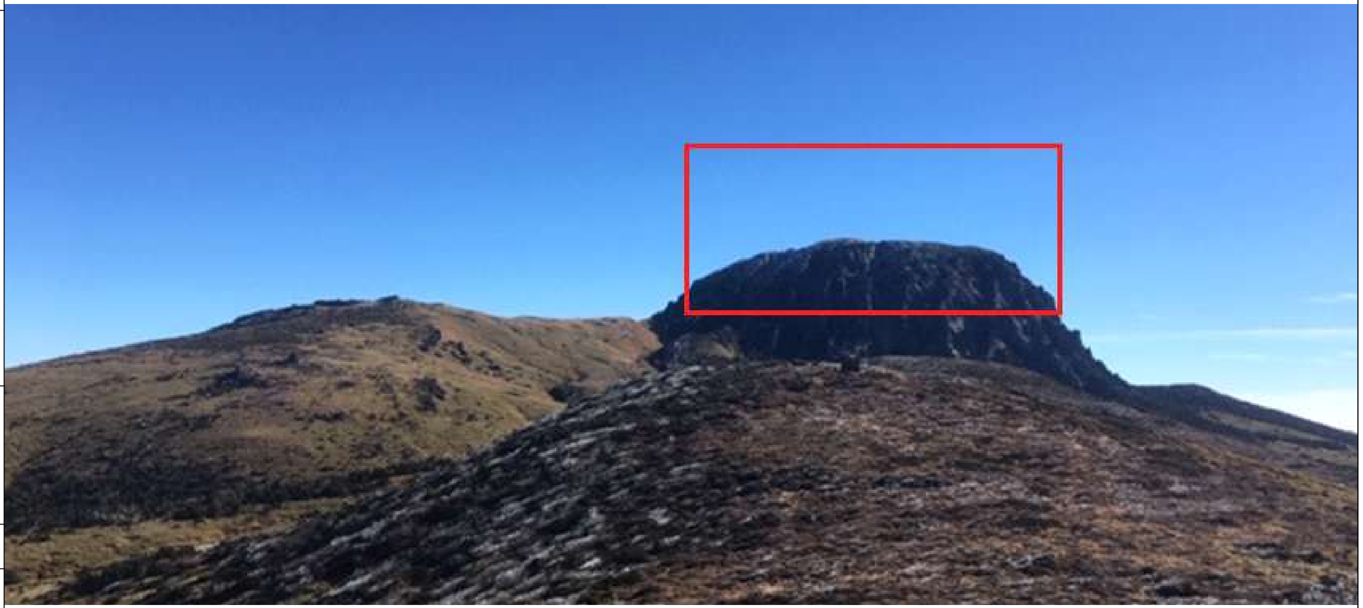

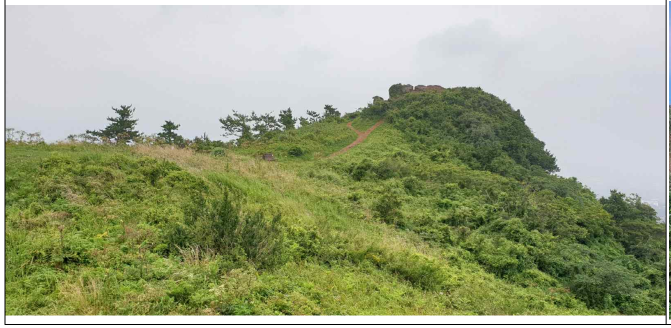

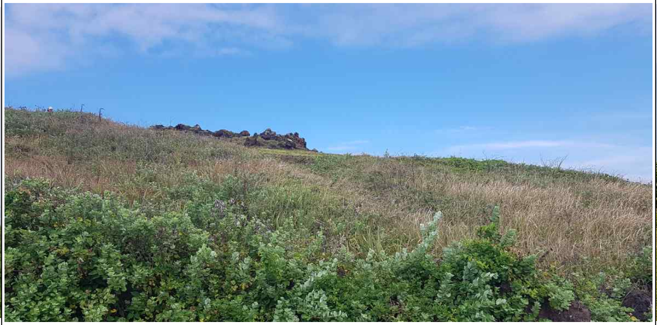

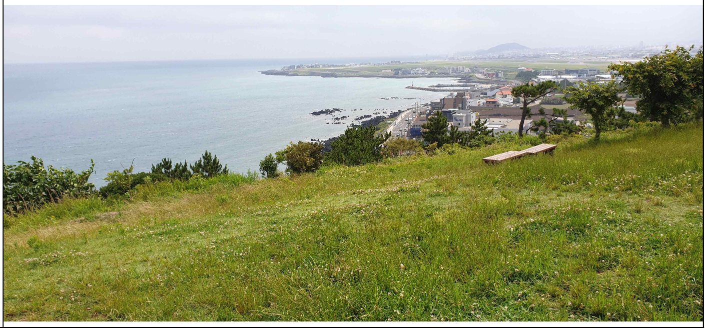

2 Shoulder

Summit	Shoulder	Slope	Mild Slope	Flat land	Channel

Landscape Conservation Value of Shoulder

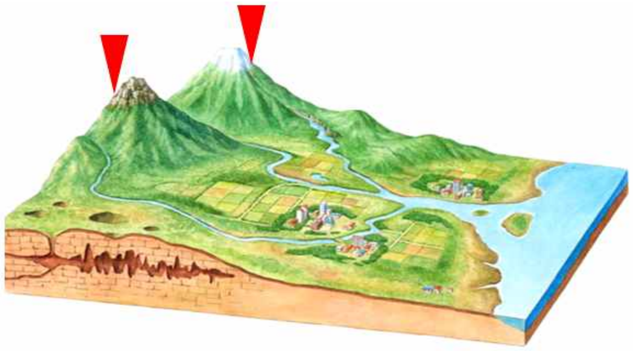

The vertical flow of water is replaced by horizontal flow as the slope increases and the flow of water inside the soil.

Very Low	Low	Normal	High	Very High
①	②	③	④	⑤

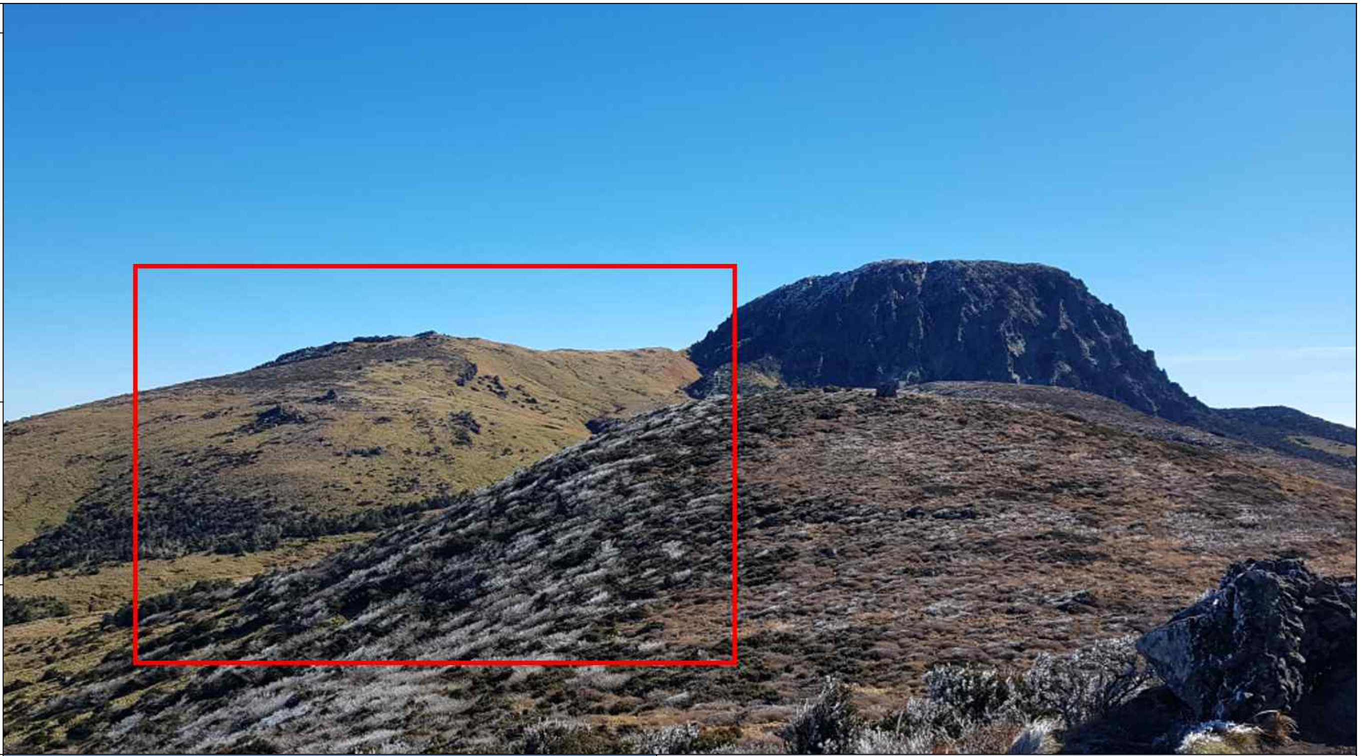

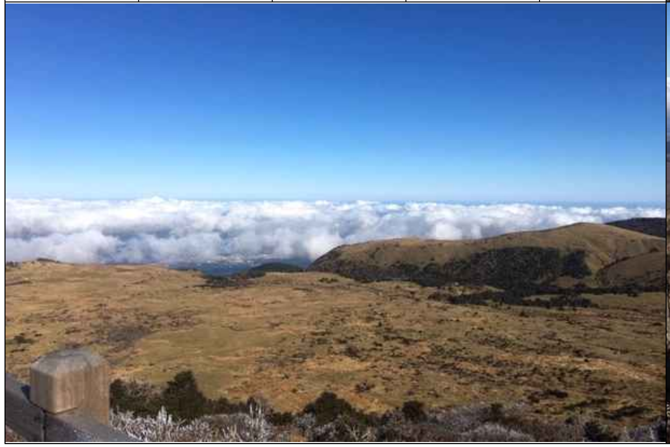

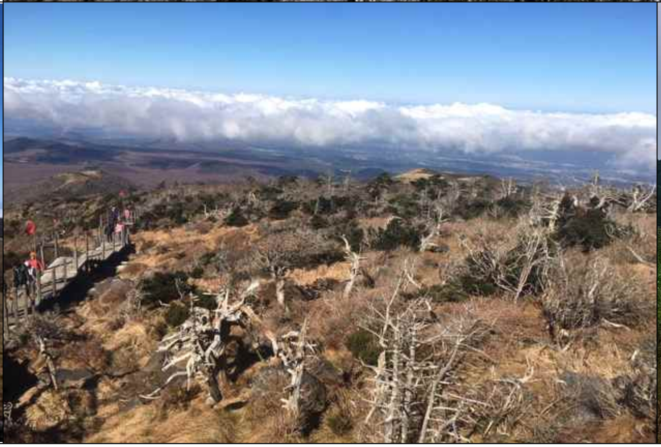

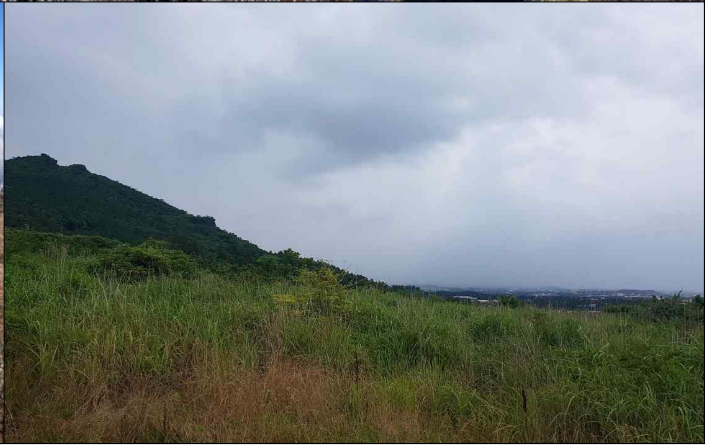

3 Slope

Summit	Shoulder	Slope	Mild Slope	Flat land	Channel

Landscape Conservation Value of Slope

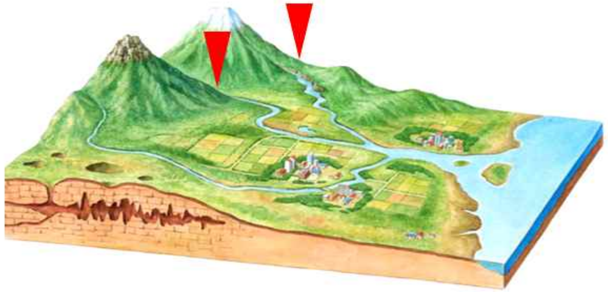

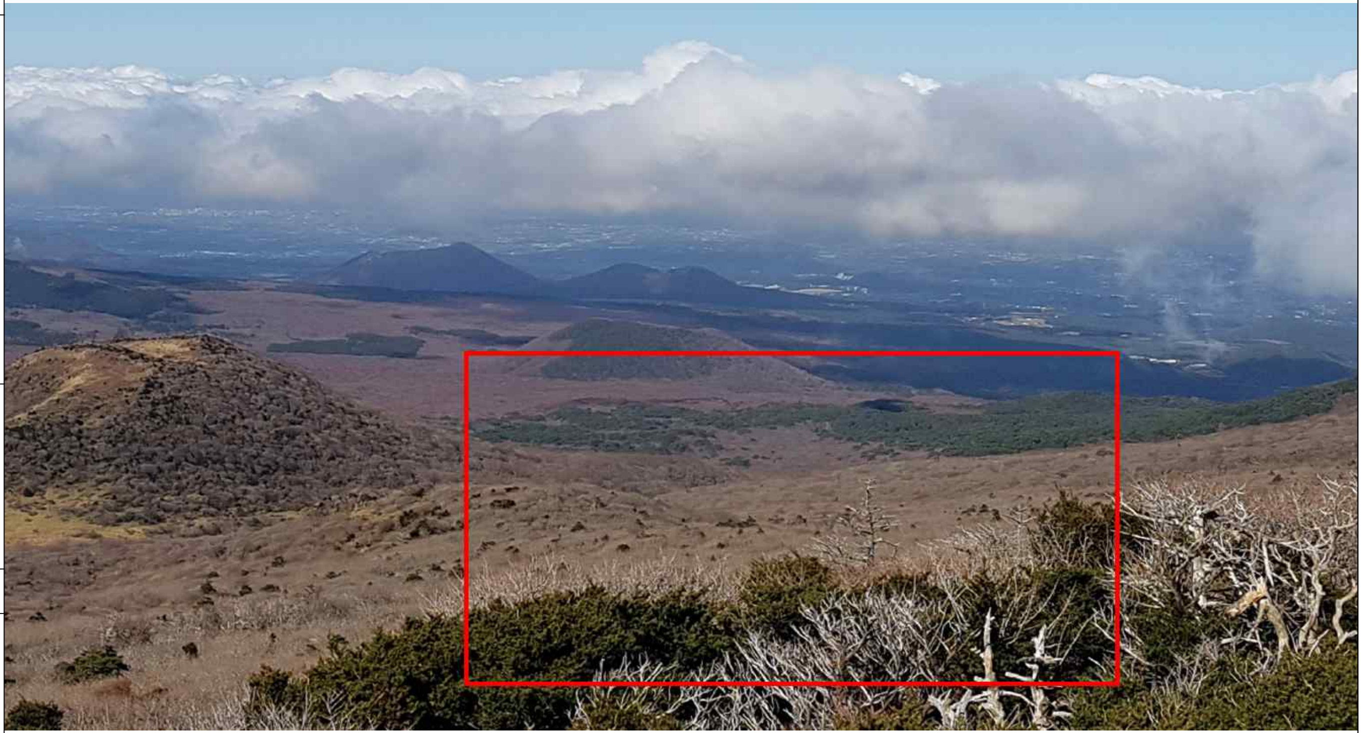

The midpoint of the upper slope, where the outflow of material prevails, and the inflow of material prevails, include major erosion, slide, creep, and movement of material within the soil layer.

Very Low	Low	Normal	High	Very High
①	②	③	④	⑤

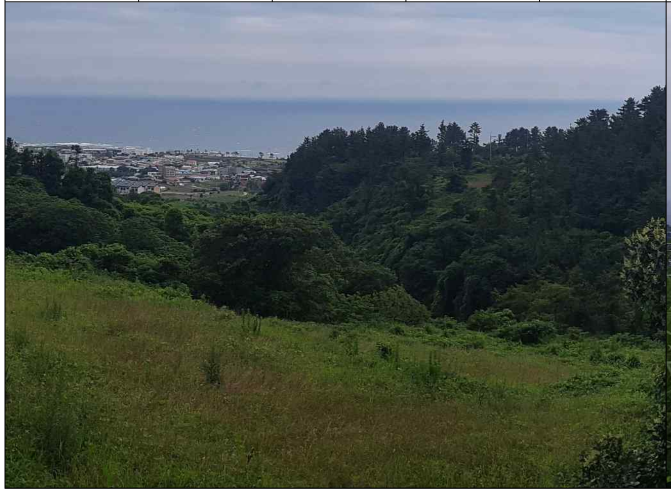

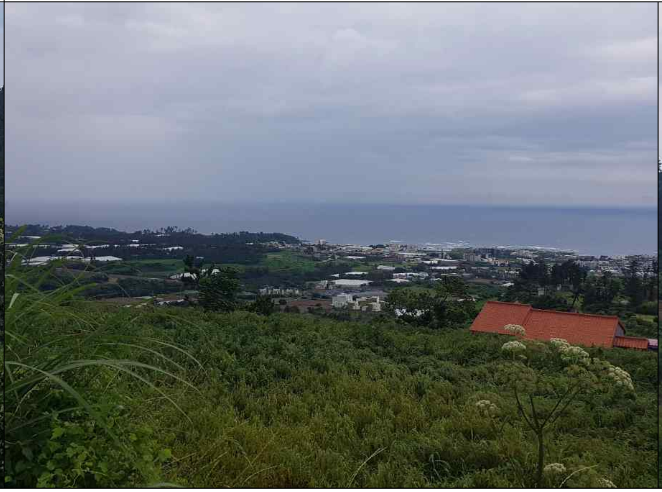

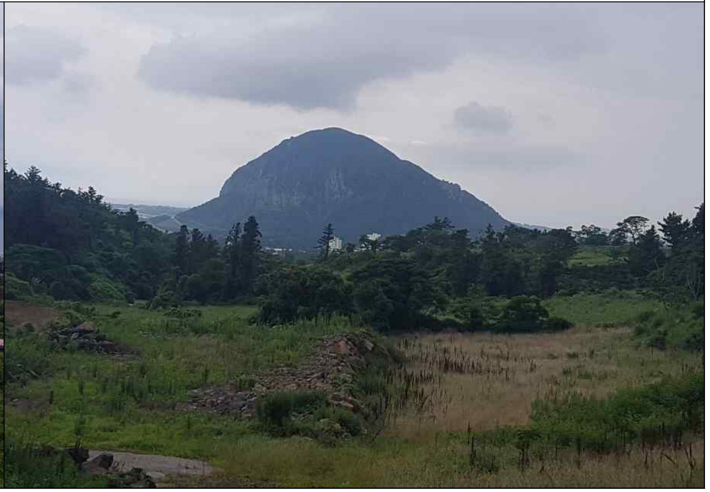

4 Mild slope

Summit	Shoulder	Slope	Mild Slope	Flat land	Channel

Landscape Conservation Value of Mild slope

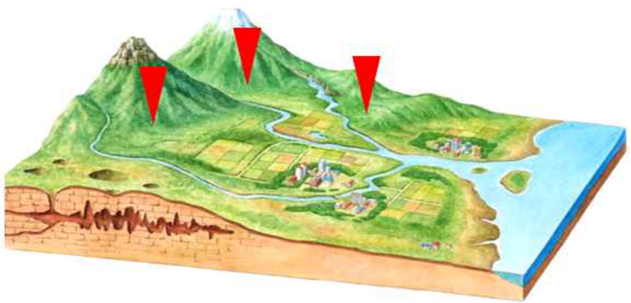

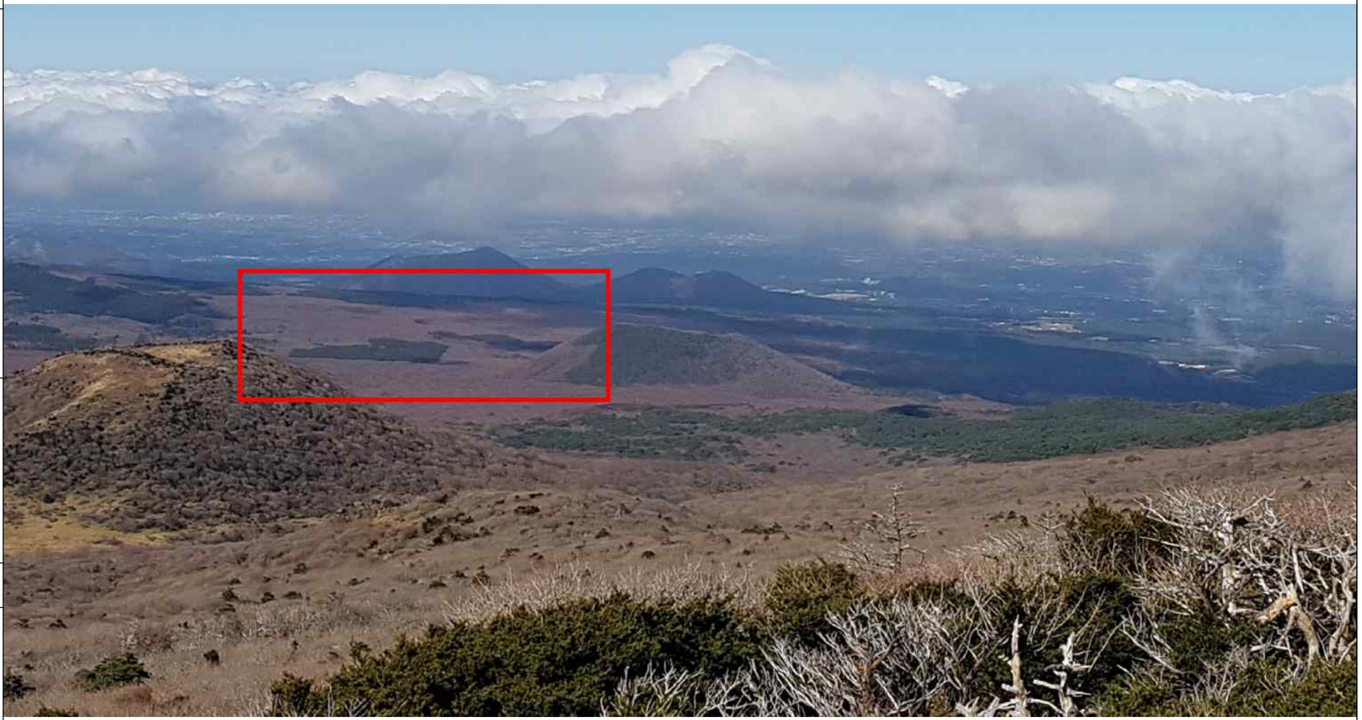

It shows the accumulation of substances from the upper soil topsoil topography surface and can observe distinct sediment layers as a result of long-term slope development.

Very Low	Low	Normal	High	Very High
①	②	③	④	⑤

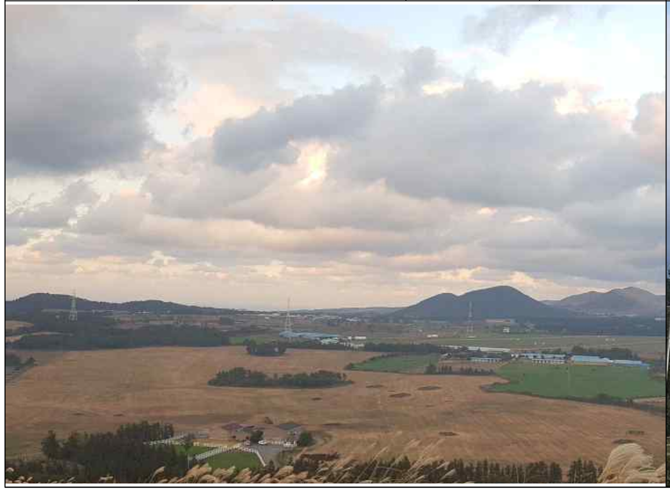

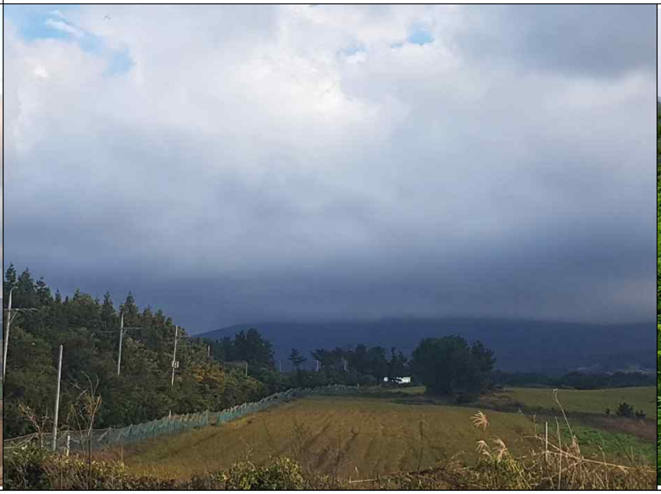

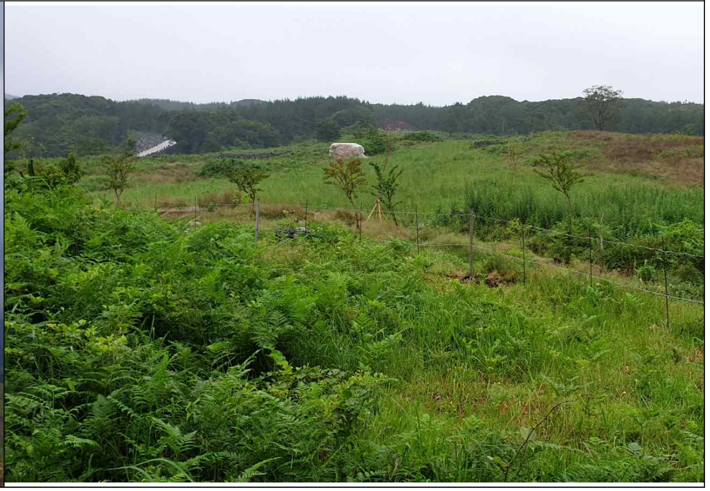

5 Flat land

Summit	Shoulder	Slope	Mild Slope	Flat land	Channel

Landscape Conservation Value of Flatland

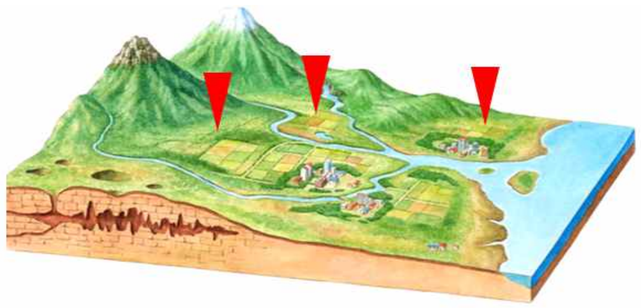

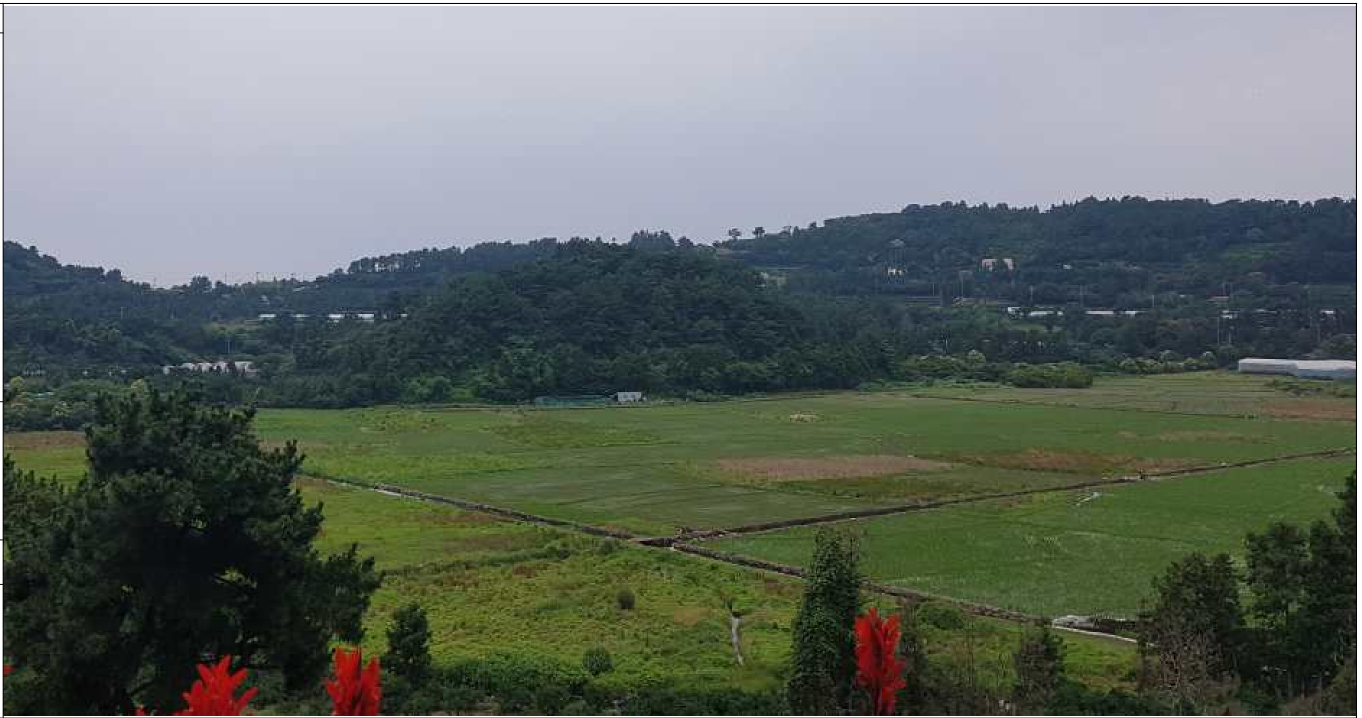

It is a topographical surface formed by the accumulation of moving materials along the stream.

Very Low	Low	Normal	High	Very High
①	②	③	④	⑤

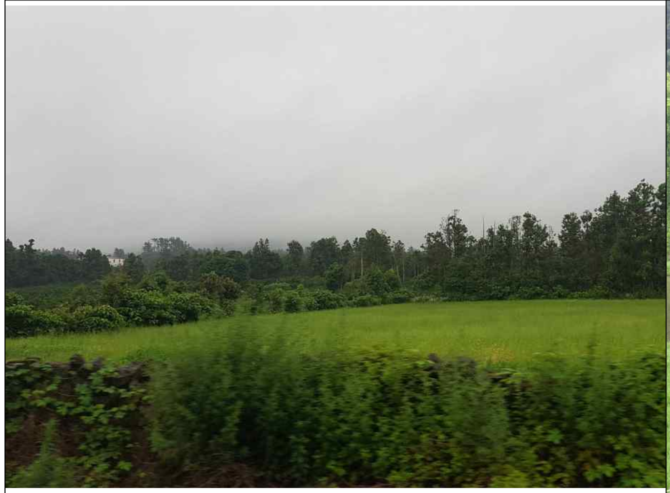

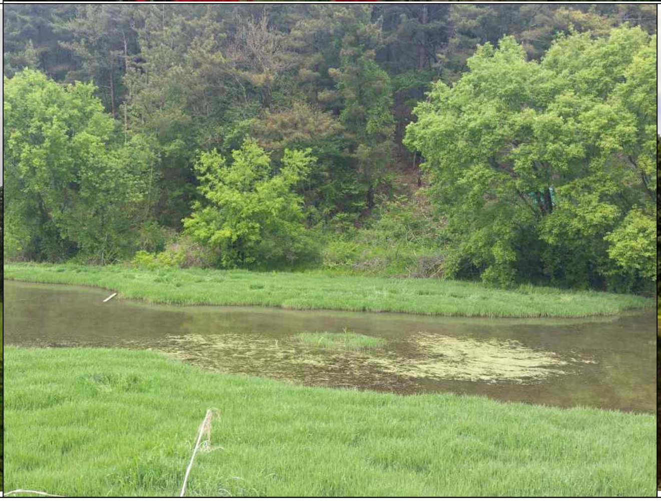

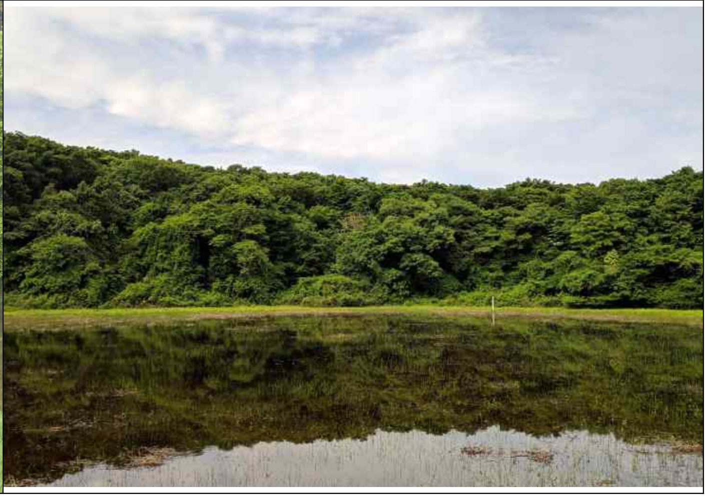

6 Channel

Summit	Shoulder	Slope	Mild Slope	Flat land	Channel

Landscape Conservation Value of Channel

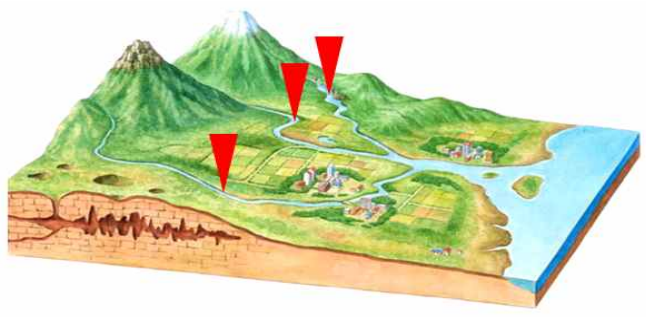

The erosion and deposition of matter by water flow. The flow of water is always present in the case of high-order streams, and the flow of water is intermittent in the case of low-order streams or a river.

Very Low	Low	Normal	High	Very High
①	②	③	④	⑤

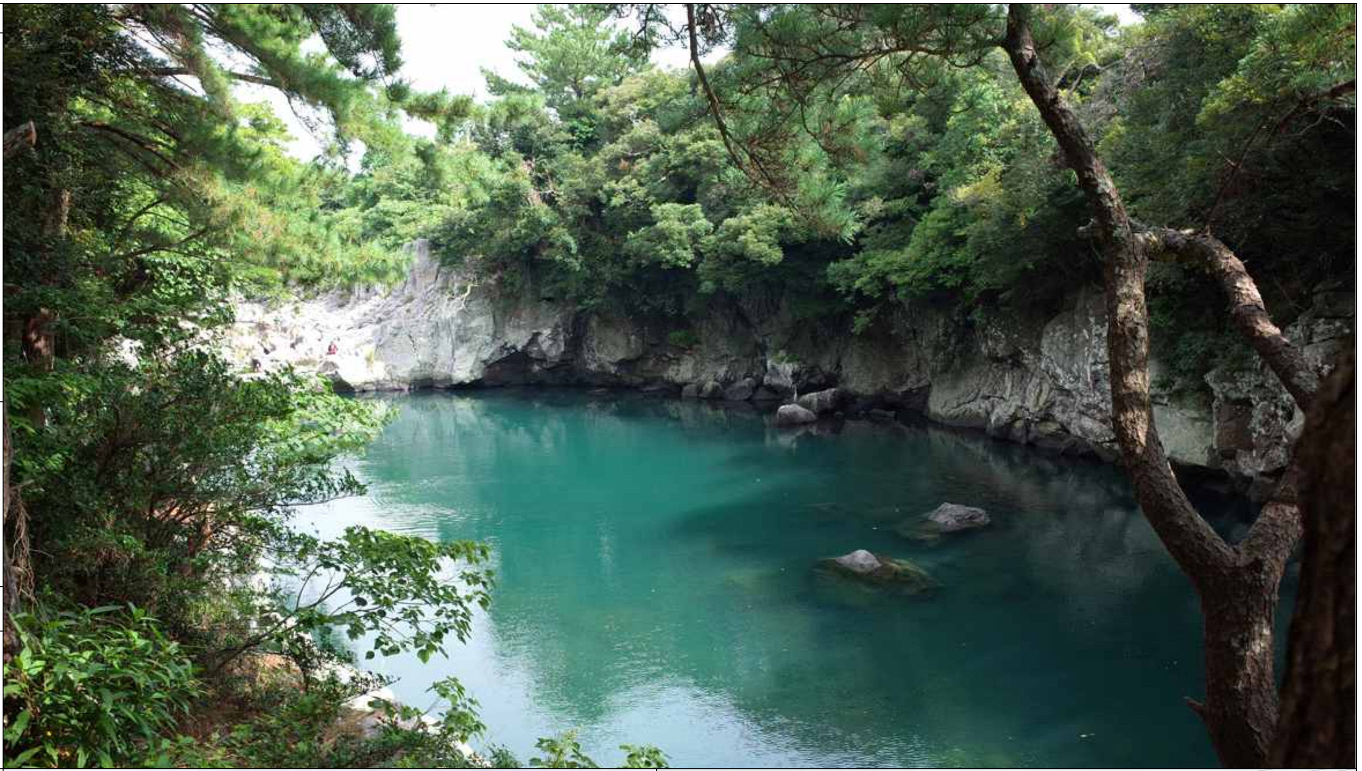

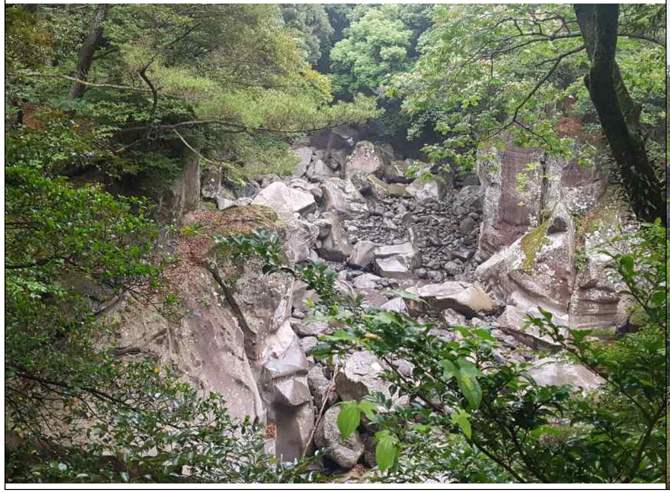

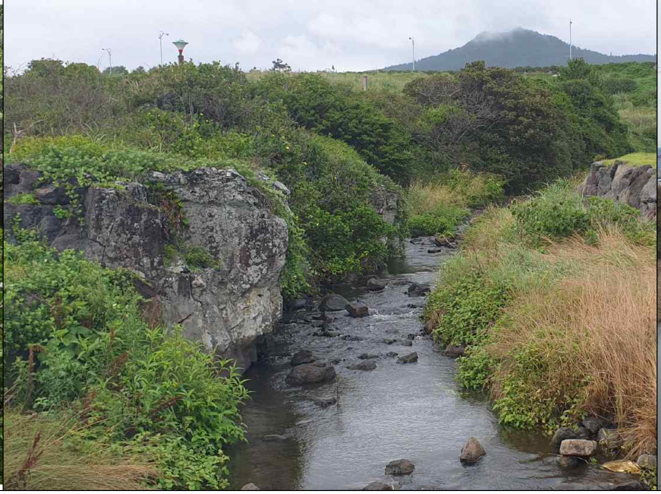

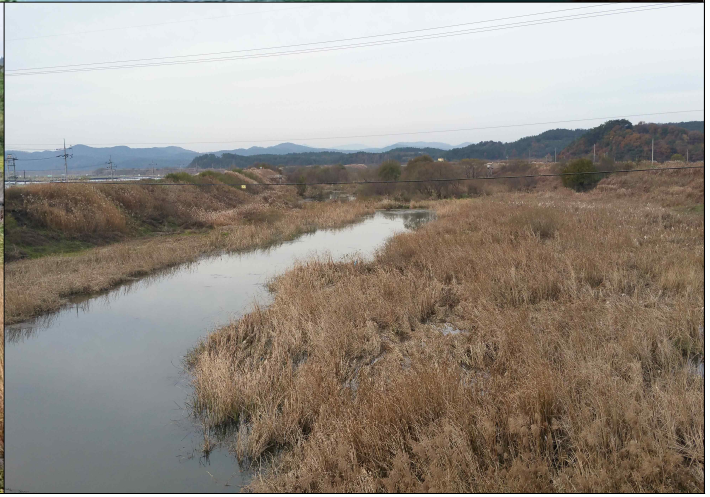

■ Land Cover

1 Developed Land

Developed Land	Agricultural Land	Forest	Grassland	Wetland	Barren land	Open water

Landscape Conservation Value of Developed land				
Includes structures such as residential, commercial and industrial facilities and transportation facilities				
Very Low	Low	Normal	High	Very High
①	②	③	④	⑤

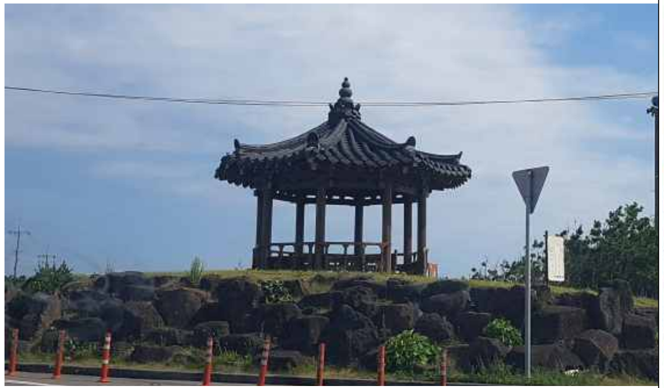

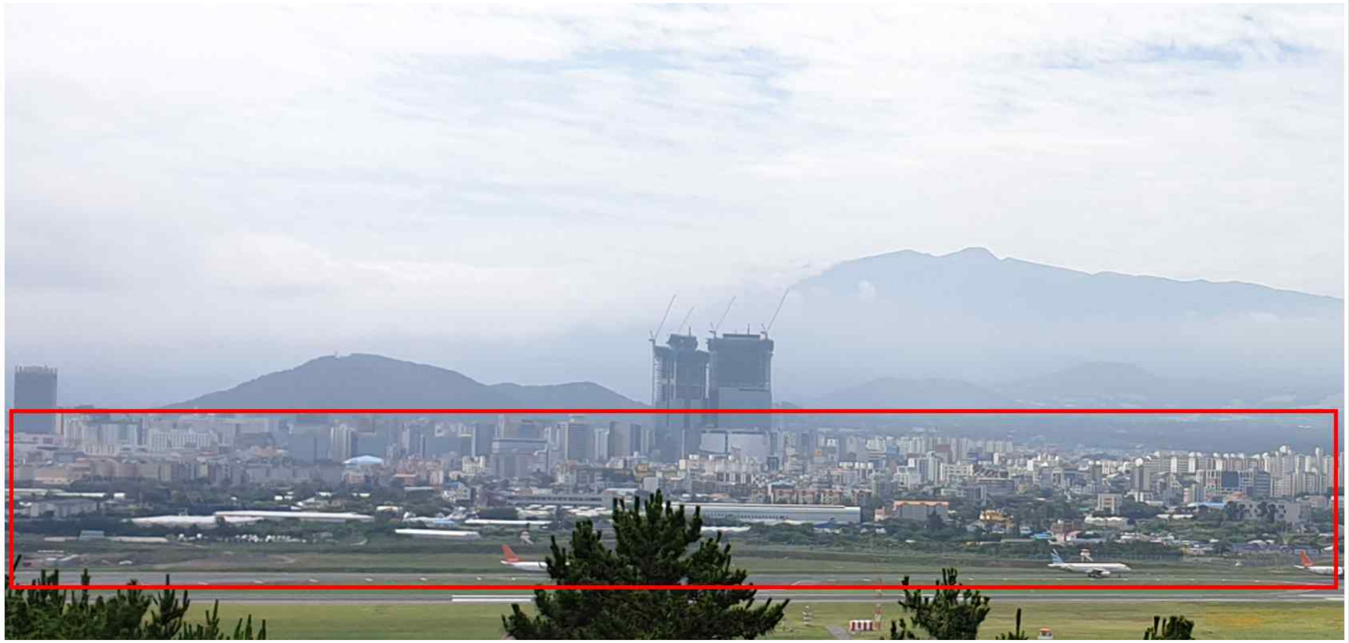

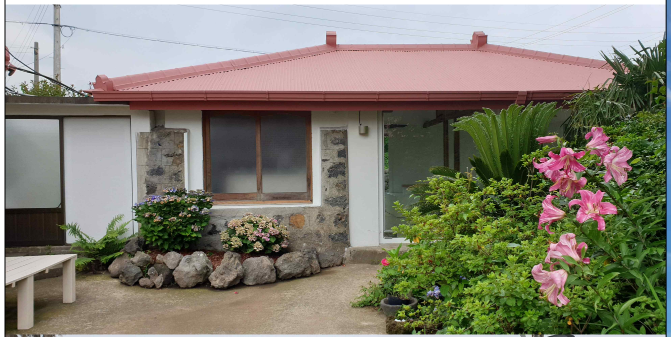

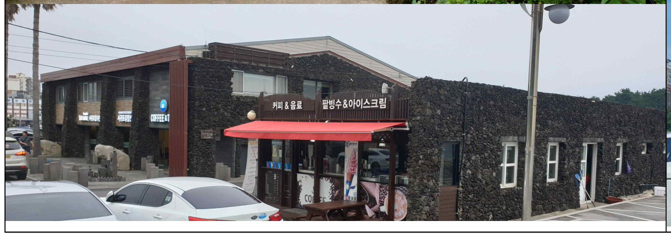

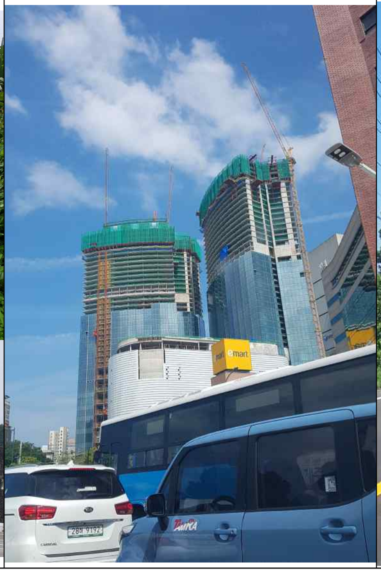

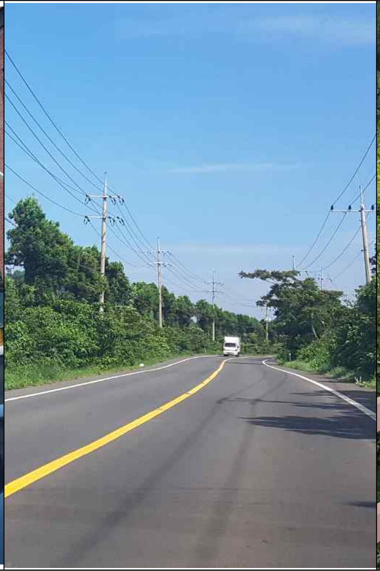

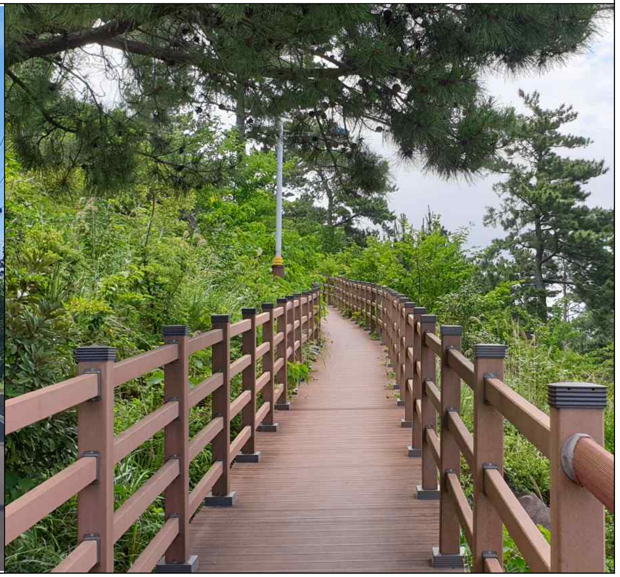

2 Agricultural land

Developed Land	Agricultural Land	Forest	Grassland	Wetland	Barren land	Open water

Landscape Conservation Value of Agricultural land				
Agricultural areas where rice paddies and fields are cultivated, and areas where fruit trees, street trees, etc. are cultivated, and facilities used for livestock and dairy farming.				
Very Low	Low	Normal	High	Very High
①	②	③	④	⑤

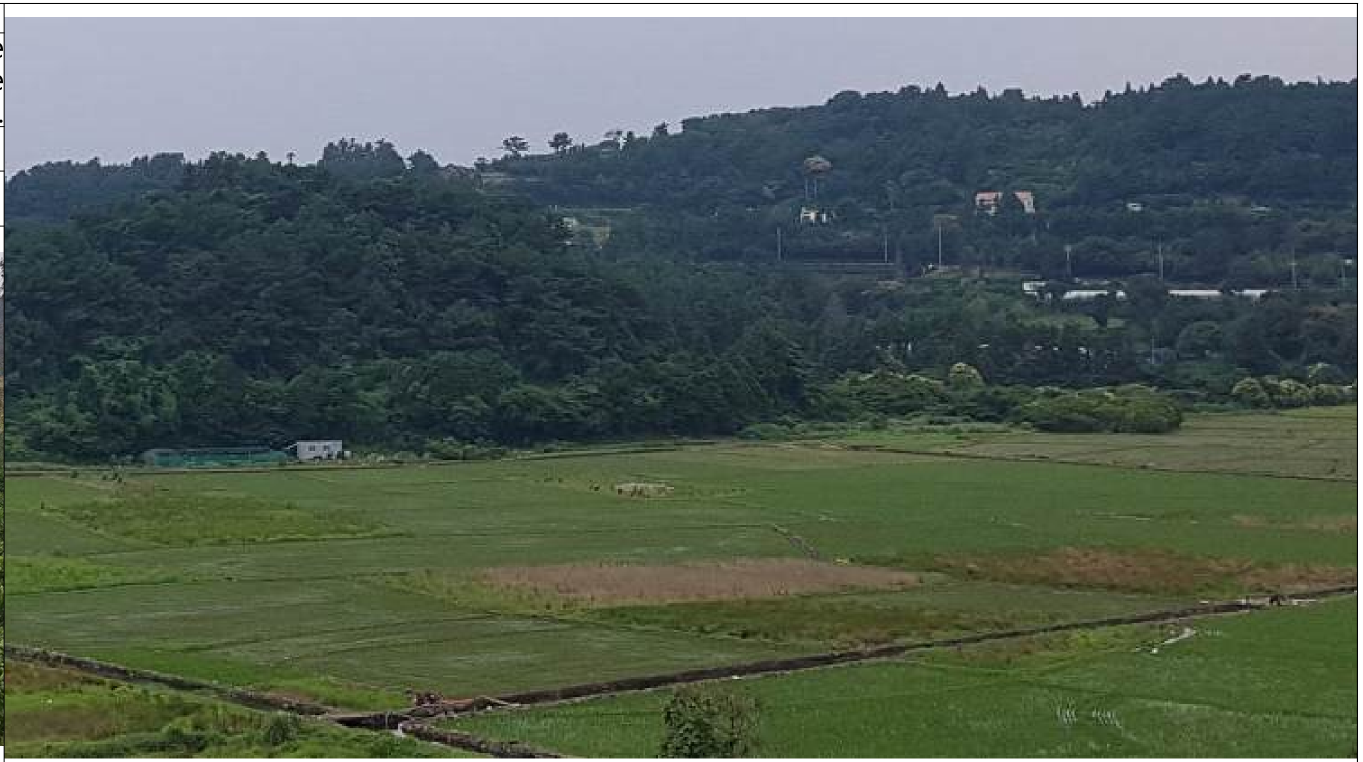

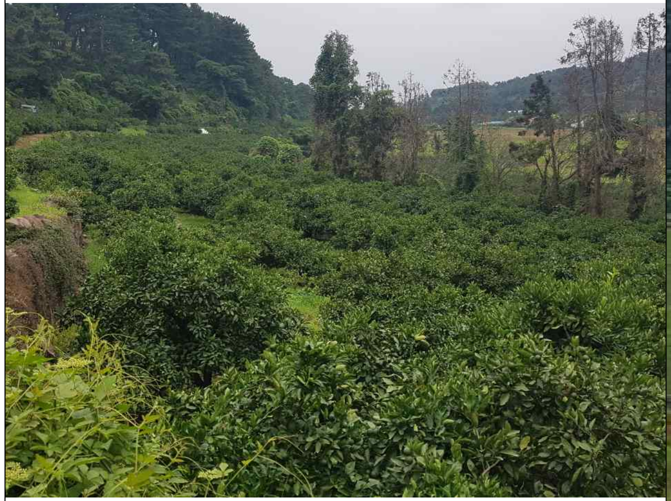

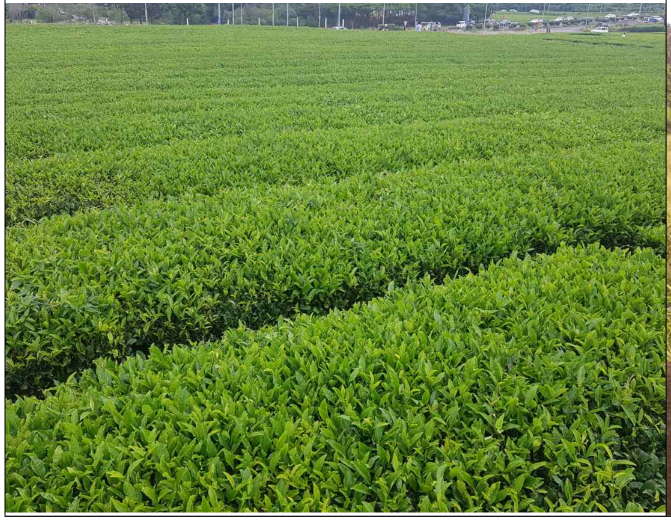

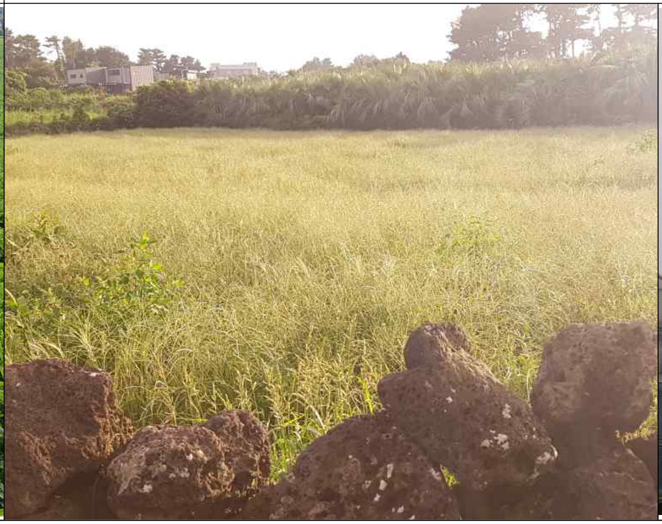

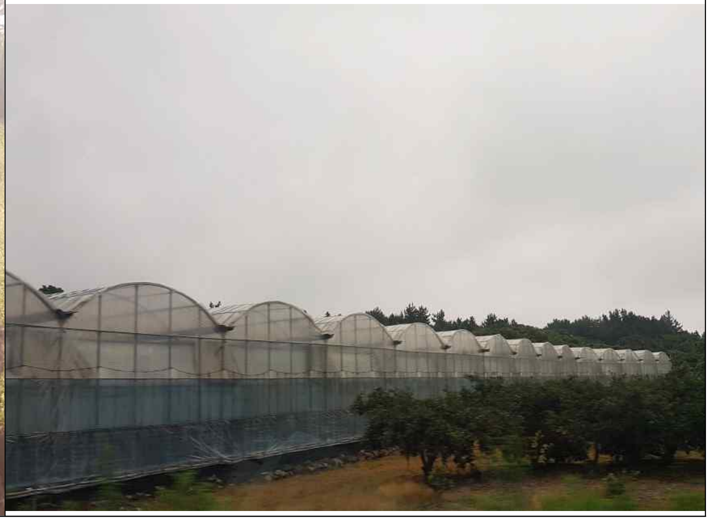

3 **Forest**

Developed Land	Agricultural Land	Forest	Grassland	Wetland	Barren land	Open water

Landscape Conservation Value of Forest				
A habitat where cluster of trees are growing				
Very Low	Low	Normal	High	Very High
①	②	③	④	⑤

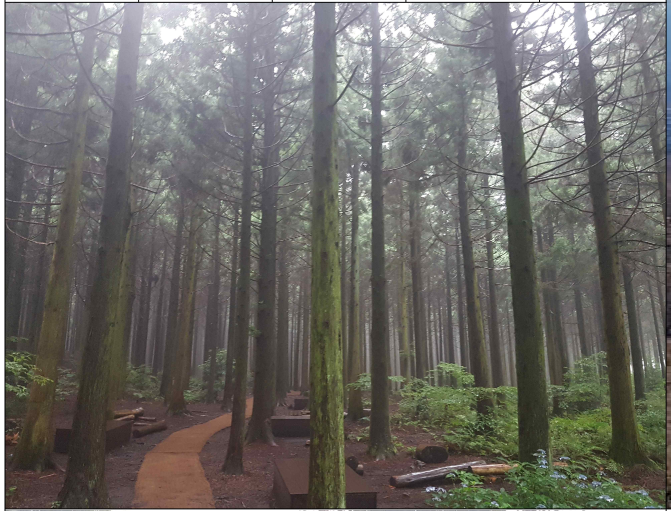

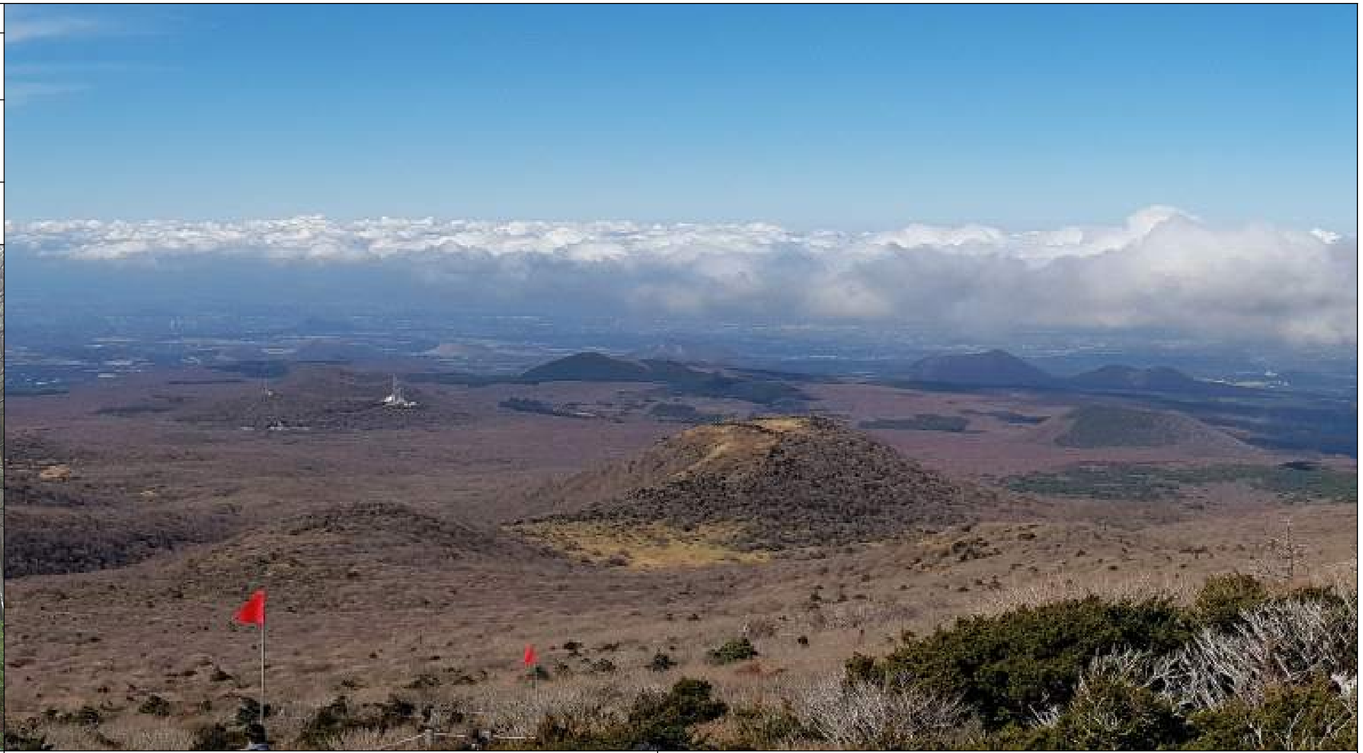

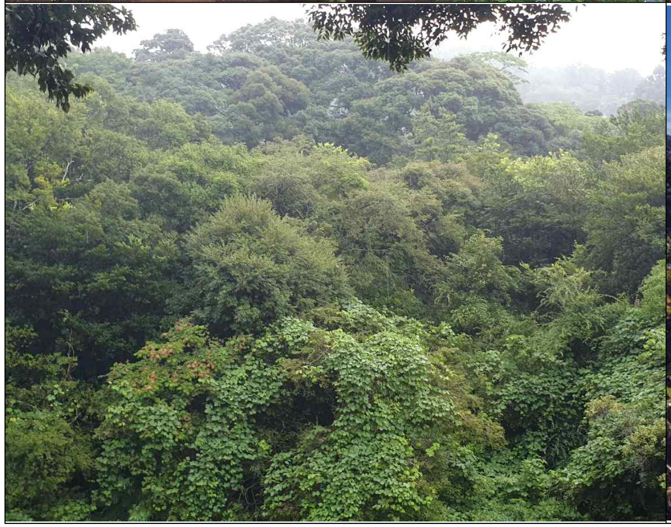

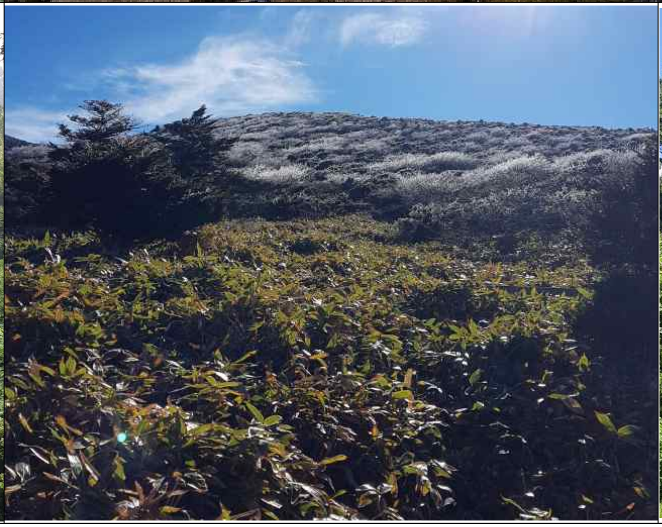

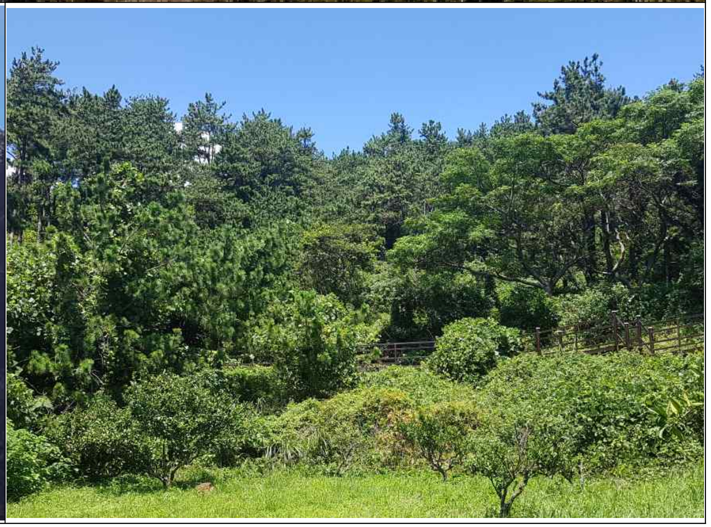

4 Grassland

Developed Land	Agricultural Land	Forest	Grassland	Wetland	Barren land	Open water

Landscape Conservation Value of Grassland

Land covered with herbaceous plants, including naturally occurring natural grasslands and artificially formed artificial grasslands.

Very Low	Low	Normal	High	Very High
①	②	③	④	⑤

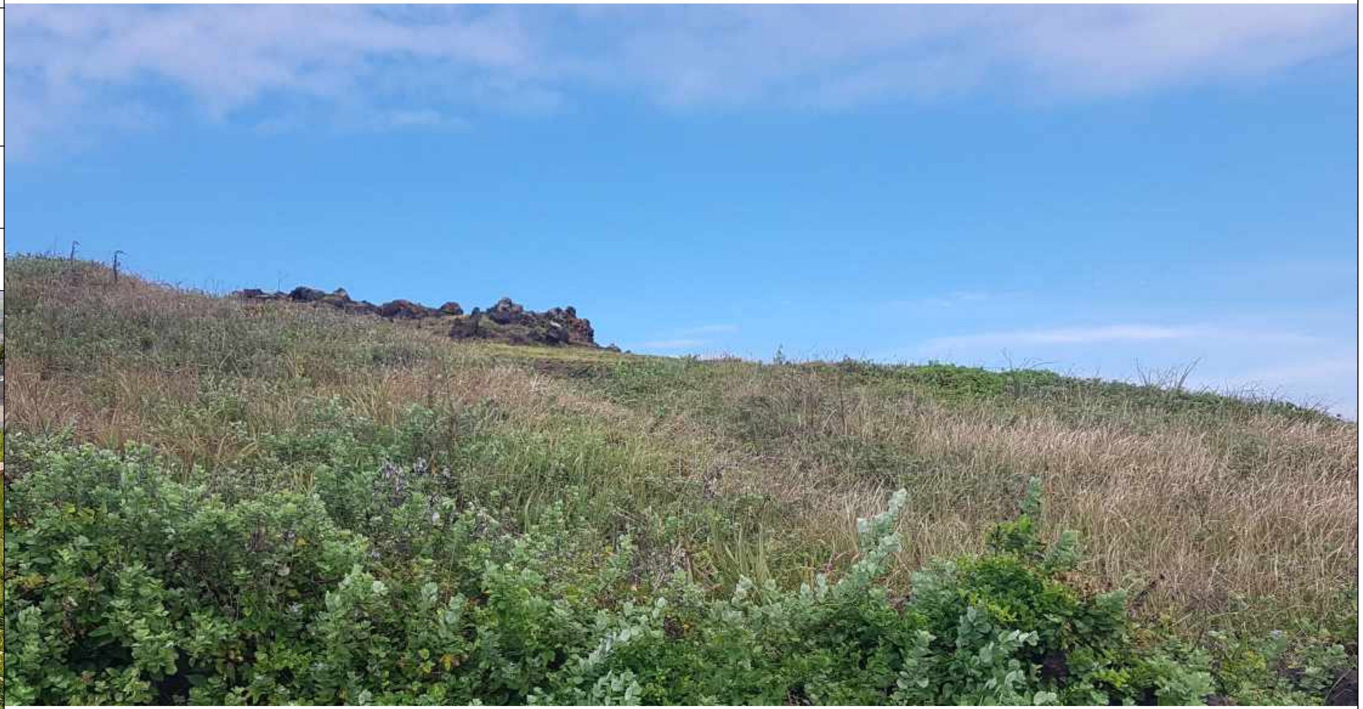

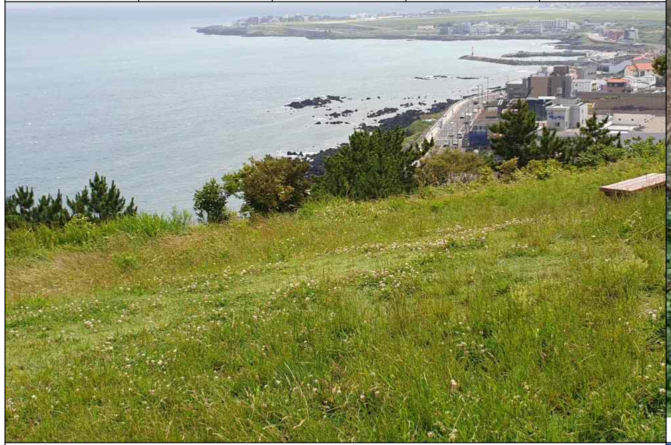

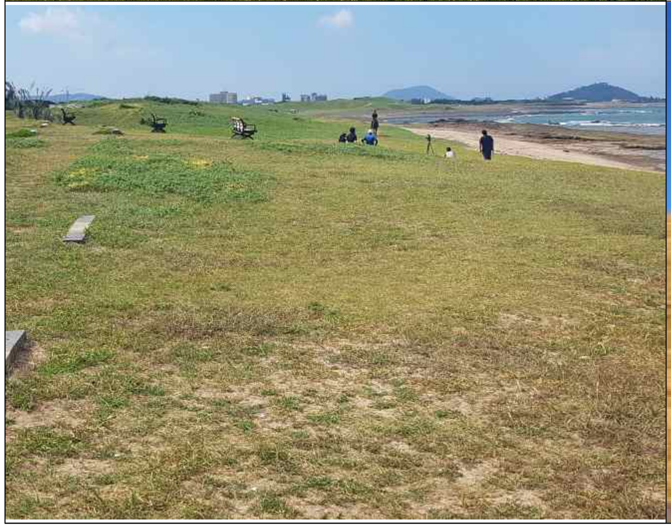

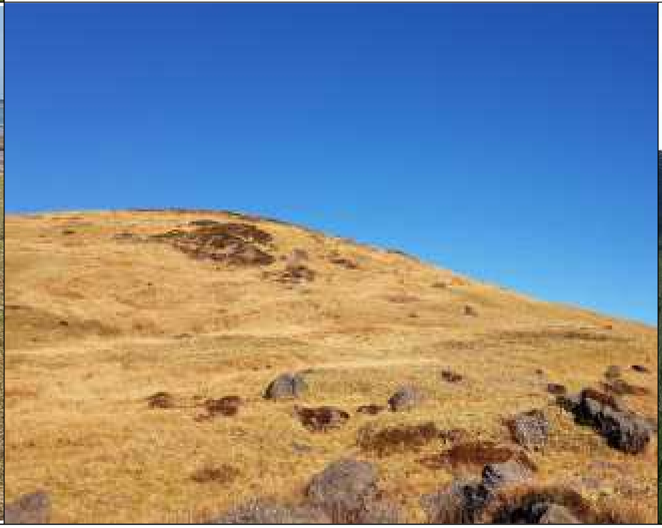

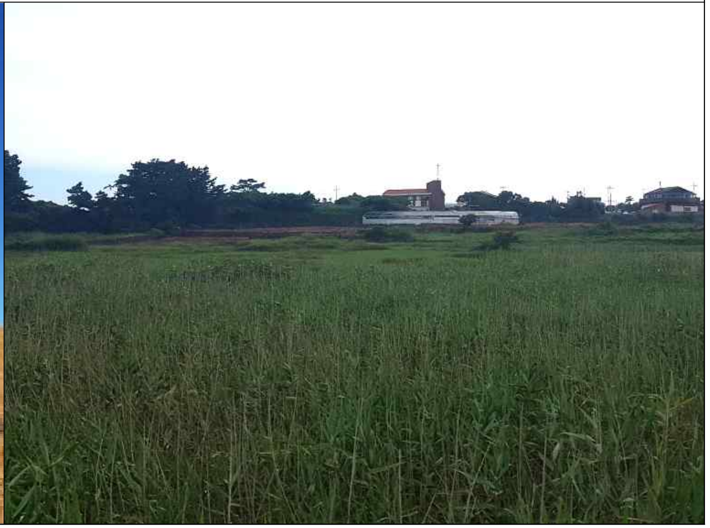

5 Wetland

Developed Land	Agricultural Land	Forest	Grassland	Wetland	Barren land	Open water

Landscape Conservation Value of Wetland				
wet and moist land that is always kept hydrated by natural environment.				
Very Low	Low	Normal	High	Very High
①	②	③	④	⑤

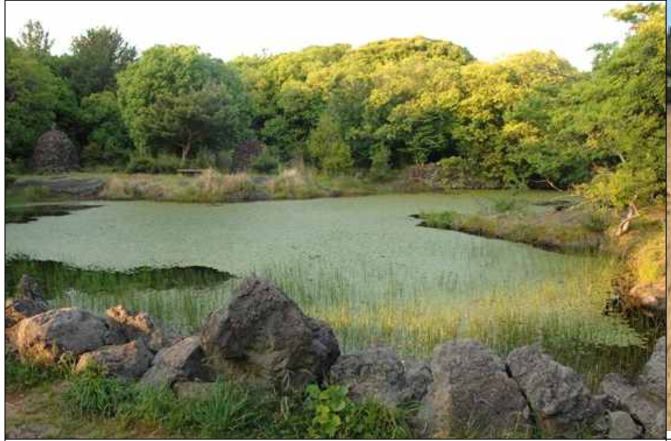

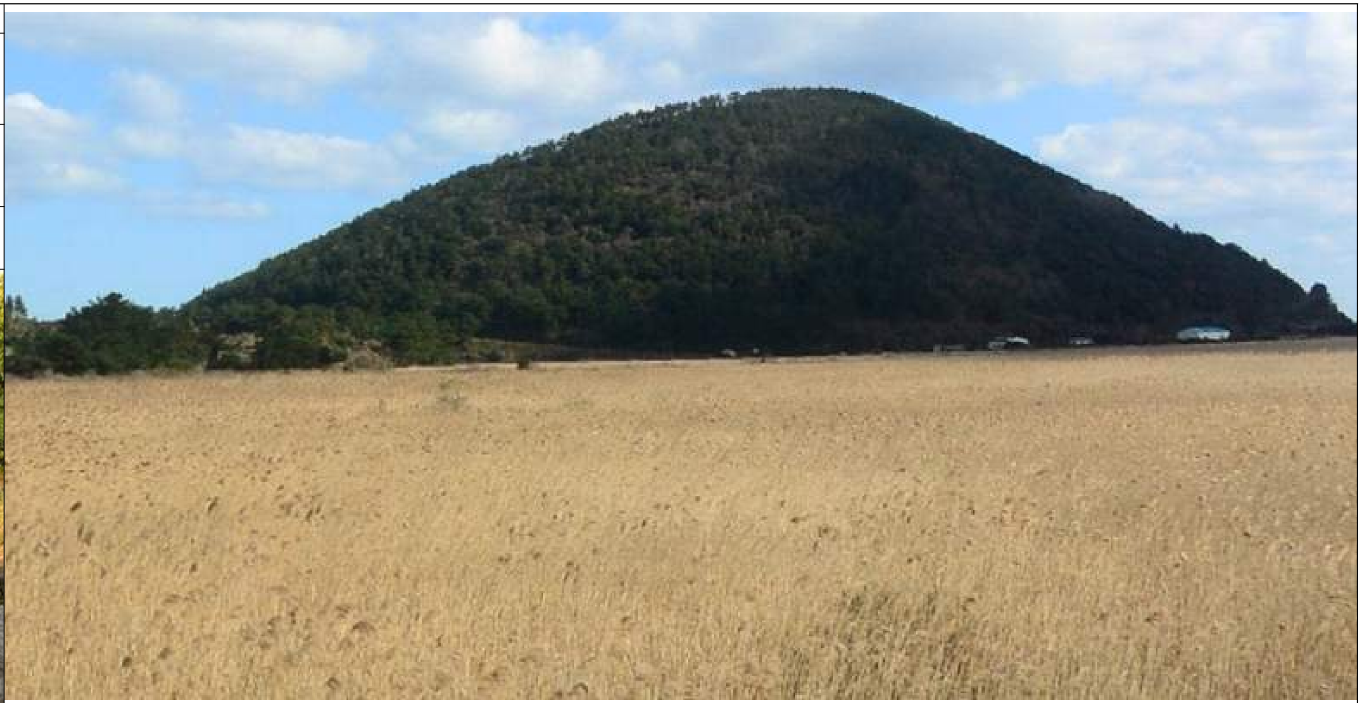

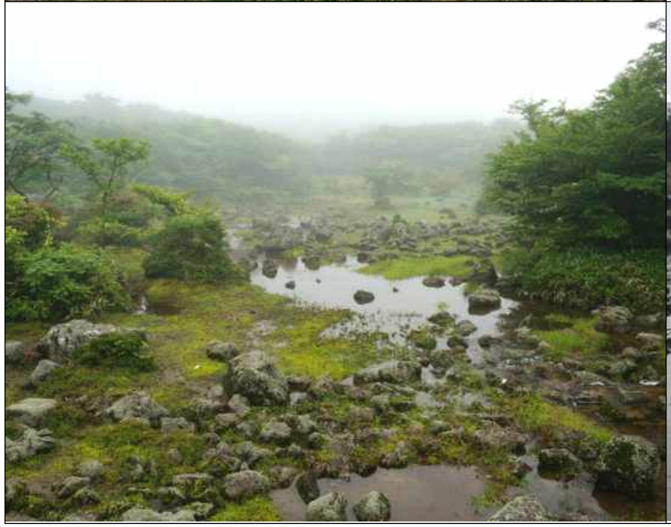

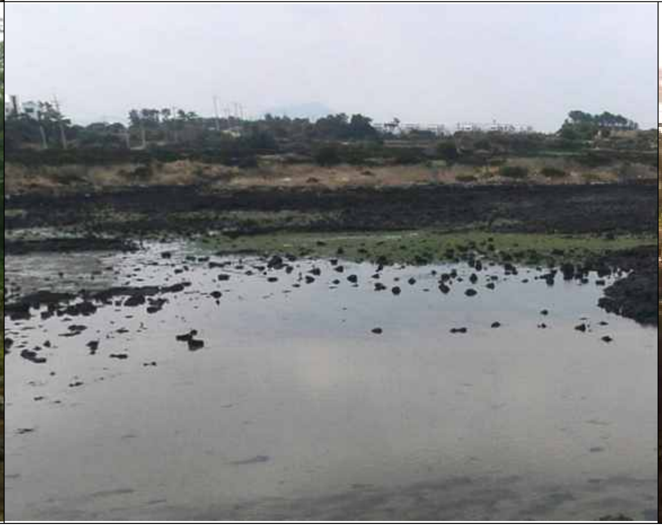

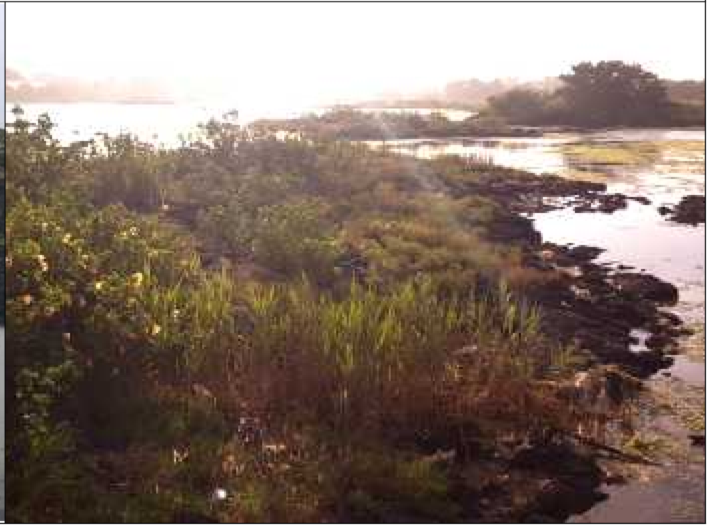

6 Barren Land

Developed Land	Agricultural Land	Forest	Grassland	Wetland	Barren land	Open water

Landscape Conservation Value of Barren land				
Bare ground with no appearance of vegetation				
Very Low	Low	Normal	High	Very High
①	②	③	④	⑤

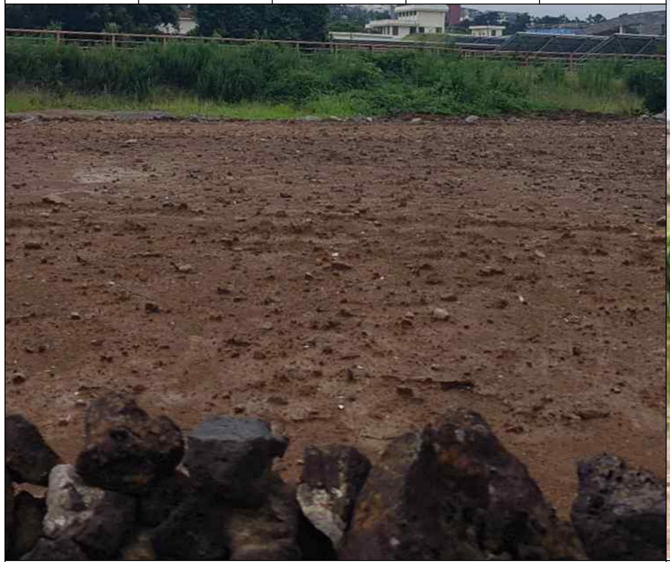

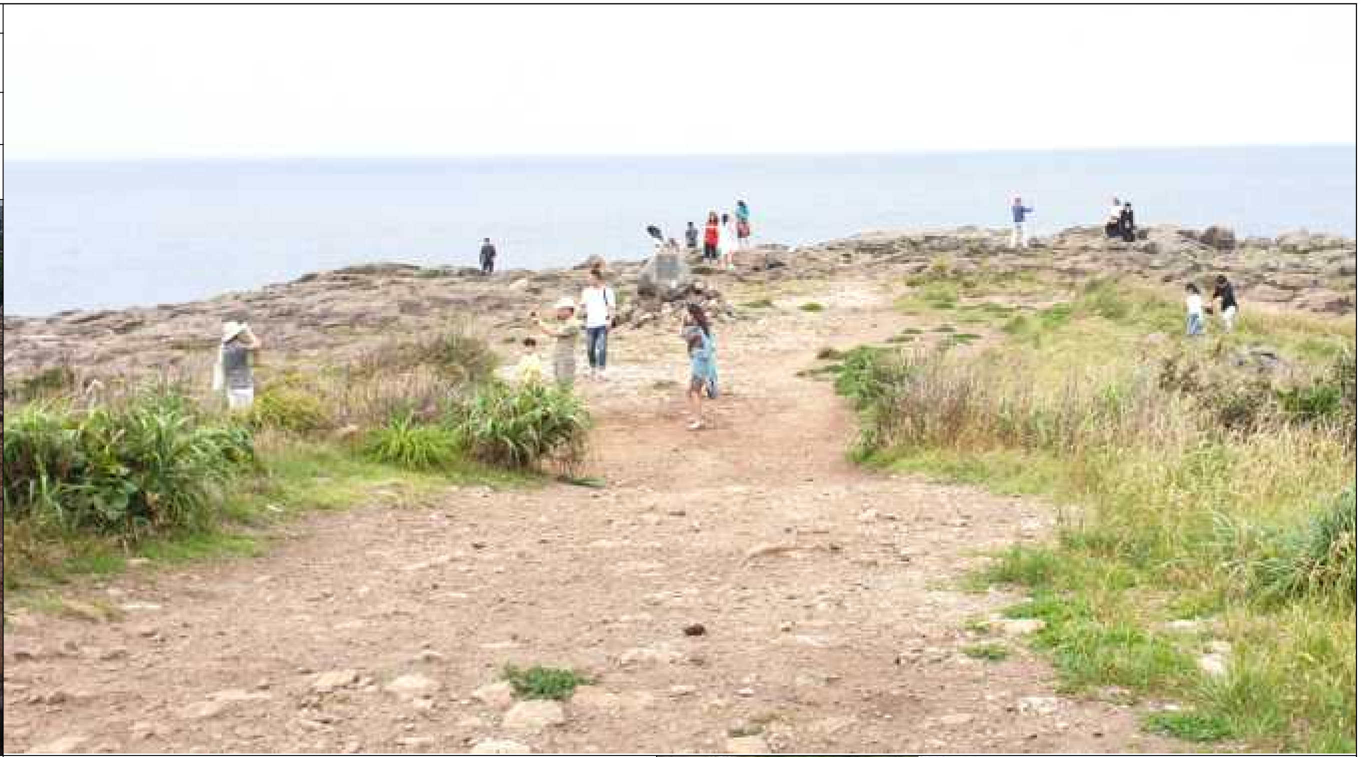

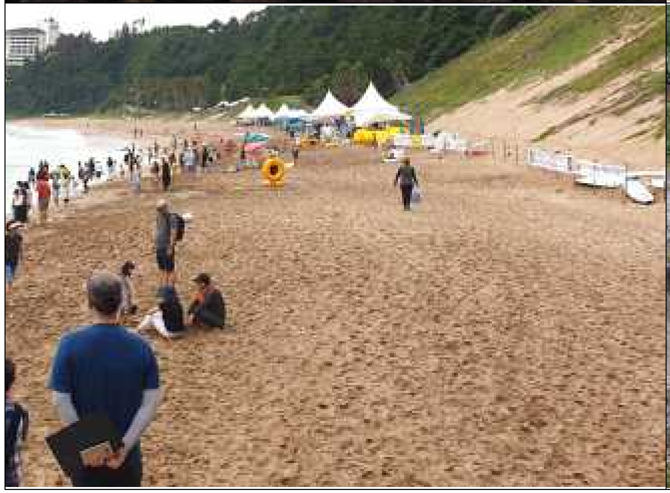

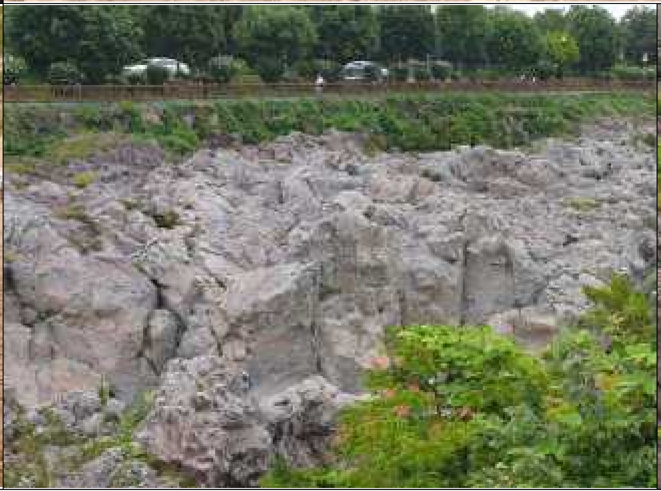

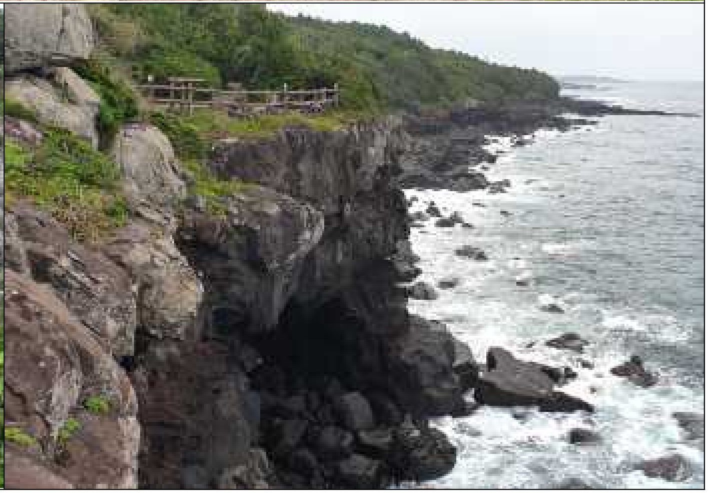

7 Open Water

Developed Land	Agricultural Land	Forest	Grassland	Wetland	Barren land	Open water

Landscape Conservation Value of Open water				
Low-lying areas of water, such as lakes, reservoirs and swamps.				
Very Low	Low	Normal	High	Very High
①	②	③	④	⑤

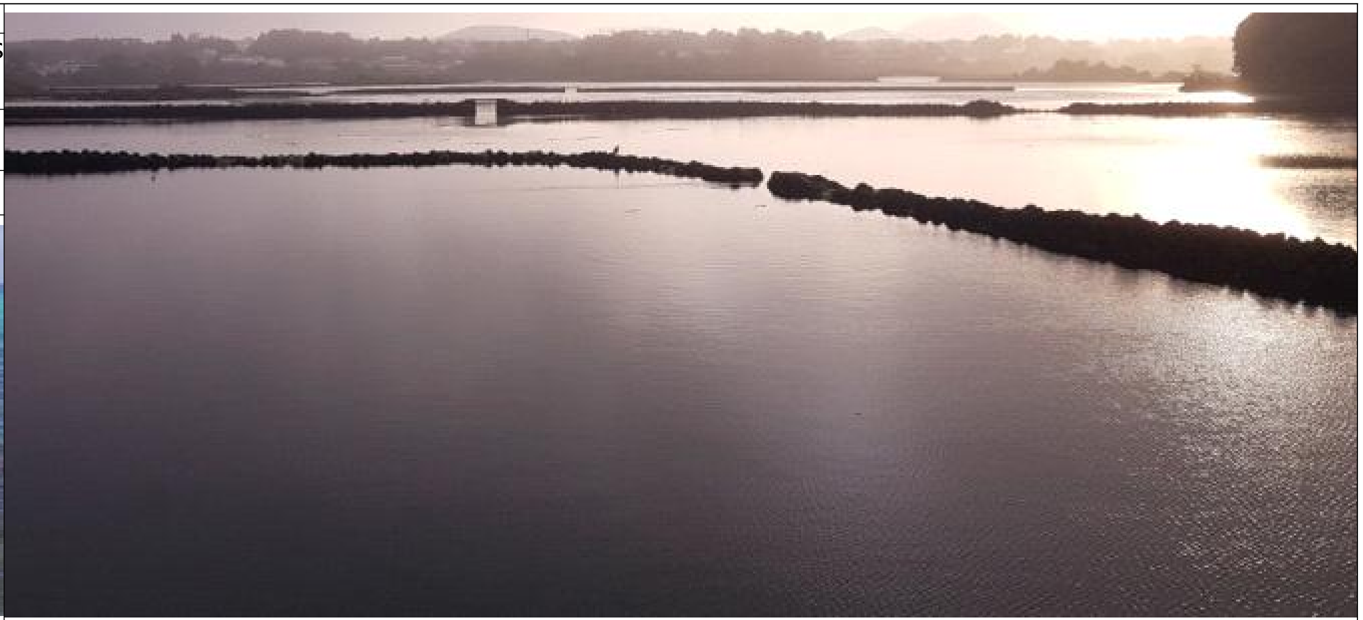
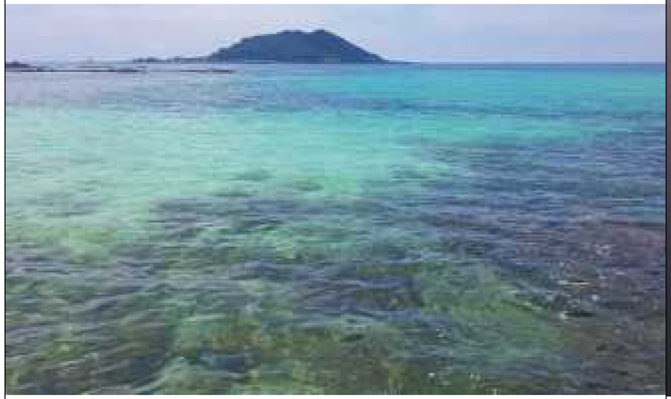
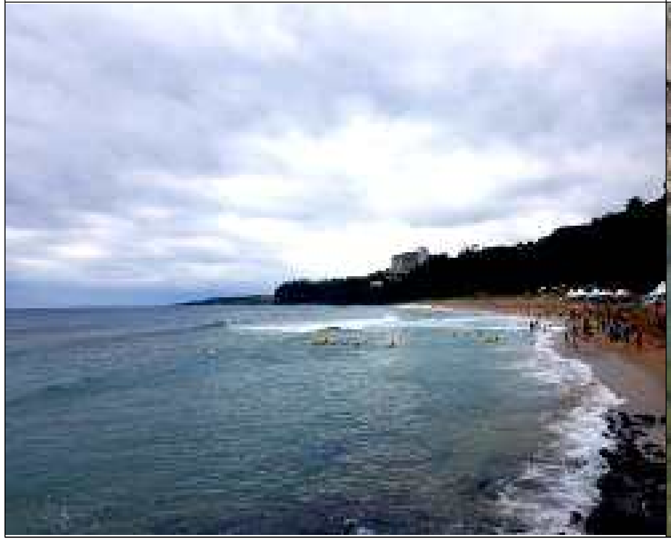
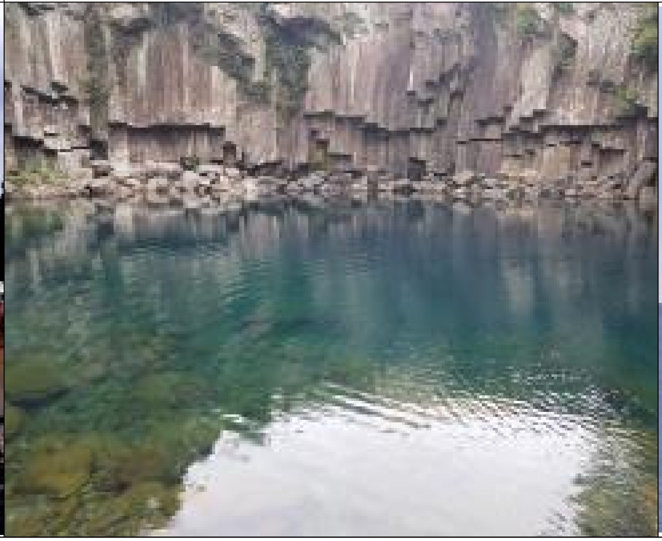
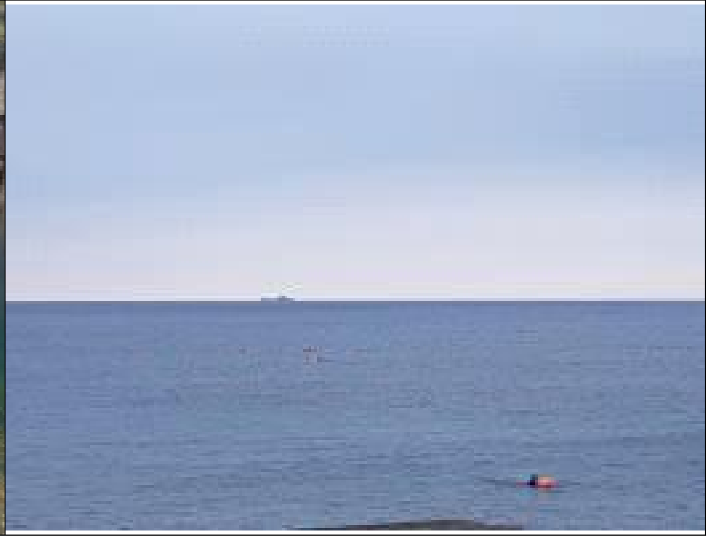

Throughout the survey different types of land cover and landform were evaluated separately. If you were to evaluate the relative importance of the landform and the land cover factors, how would you rate their relative importance? Please check one blank..

Evaluation Standard A	A is more important			Even	B is more important			Evaluation Standard B
	Much More	More	Slightly More		Slightly More	More	Much More	
Landform								Land Cover

Personal Information

Name	
Age	20s☐ 30s☐ 40s☐ 50s☐ 60s☐
Major	
Education	Bachelor☐ Master☐ Ph.D☐
Work Experience	( )years
Contact	Affiliation:
	Address:
	E-mail address:

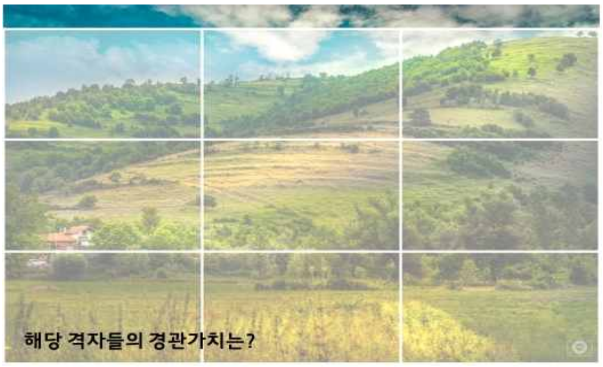

Thank you for your Participation.

This the 1st part of the survey and further later you will be asked to participate in 2nd part of the survey.

Please send your response to baysokjun@nie.re.kr within two to three days.
