# Supplementary material for: Development of landscape conservation value map of Jeju island, Korea for integrative landscape management and planning using conservation value of landscape typology
Source: PeerJ. 2021 Jun 1;9:e11449. doi: 10.7717/peerj.11449 (PMC8176906; doi:10.7717/peerj.11449)
Supplement: Supplemental Information 6 [file peerj-09-11449-s006.pdf]

2nd Expert Survey on Landscape Conservation Value

Resetting landscape conservation value of land cover and landform type combination

Please rate a landscape conservation value of land cover and landform type combination based on 6 factors(Representativeness, specificity, diversity, rarity, irreproducibility and educational value) based on your expert knowledge and experience.

For each landscape types the median value of the 1st survey results derived from 13 experts are displayed(↔) on the top of the response scale, followed by the 1st survey results of individuals are marked(√) below as a reference.

※ 'Slope' is added as a new type of landform to this survey. Please rate conservation value of 'Slope' combined with 7 different land covers.

Please give an appropriate conservation value of each landscape types from scale of 1 to 10 in 're-evaluation [ ]' row

<Summit>

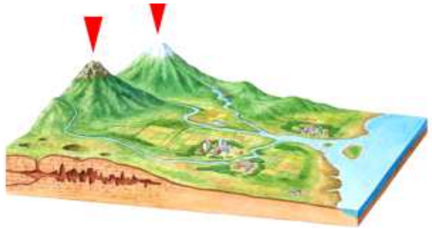

	습지<br>5.58	수역<br>5.50	산림지<br>5.12	초지<br>4.75	농업지역<br>3.96	시가화 건조지역<br>3.35	나지(암반포함)<br>3.06
봉우리<br>5.19	28.96<br>10점	28.56<br>10점	26.56<br>9점	24.66<br>8점	20.57<br>6점	17.37<br>4점	15.88<br>3점
하천부지<br>4.62	25.74<br>9점	25.38<br>9점	23.61<br>8점	21.92<br>7점	18.28<br>5점	15.44<br>3점	14.11<br>2점
상부<br>4.19	23.38<br>7점	23.06<br>7점	21.45<br>6점	19.91<br>5점	16.61<br>4점	14.03<br>2점	12.82<br>2점
구릉지<br>3.59	20.02<br>6점	19.74<br>5점	18.36<br>5점	17.05<br>4점	14.22<br>2점	12.01<br>1점	10.98<br>1점
급경사지<br>-	-	-	-	-	-	-	-

Explanation: A flat surface that appears at the top of a mountain, with little slope. Because it is located at the top of the mountain, the inflow of material from the surroundings is limited, and the outflow of energy and material into the lower slope is limited due to the low surface gradient.

1. Conservation value of Wetland formed on top of summit

Low	13 Experts(Median)	1	2	3	4	5	6	7	8	↔ 9	10	High
	1st Survey Result(individual)											
	Re-evaluation											

2. Conservation value of openwater formed on top of summit

Low	13 Experts(Median)	1	2	3	4	5	6	7	8	9	↔ 10	High
	1st Survey Result(individual)											
	Re-evaluation											

3. Conservation value of forest formed on top of summit

Low	13 Experts(Median)	1	2	3	4	5	6	7	8	9	↔ 10	High
	1st Survey Result(individual)											
	Re-evaluation											

4. Conservation value of grassland formed on top of summit

Low	13 Experts(Median)	1	2	3	4	↔ 5	6	7	8	9	10	High
	1st Survey Result(individual)											
	Re-evaluation											

5. Conservation value of Agricultural land formed on top of summit

Low	13 Experts(Median)	1	2	3	4	5	↔ 6	7	8	9	10	High
	1st Survey Result(individual)											
	Re-evaluation											

6. Conservation value of developed land formed on top of summit

Low	13 Experts(Median)	1	2	↔ 3	4	5	6	7	8	9	10	High
	1st Survey Result(individual)											
	Re-evaluation											

7. Conservation value of barrenland(rock) formed on top of summit

Low	13 Experts(Median)	1	2 ↔	3	4	5	6	7	8	9	10	High
	1st Survey Result(individual)											
	Re-evaluation											

<Shoulder>

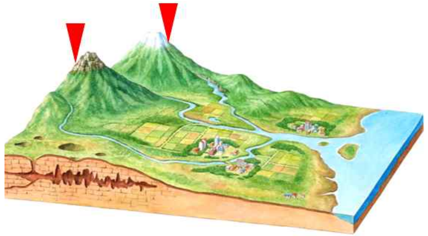

	습지<br>5.58	수역<br>5.50	산림지<br>5.12	초지<br>4.75	농업지역<br>3.96	시가화<br>건조지역<br>3.35	나지<br>(암반포함)<br>3.06
봉우리<br>5.19	28.96<br>**10점**	28.56<br>**10점**	26.56<br>**9점**	24.66<br>**8점**	20.57<br>**6점**	17.37<br>**4점**	15.88<br>**3점**
하천부지<br>4.62	25.74<br>**9점**	25.38<br>**9점**	23.61<br>**8점**	21.92<br>**7점**	18.28<br>**5점**	15.44<br>**3점**	14.11<br>**2점**
상부<br>4.19	23.38<br>**7점**	23.06<br>**7점**	21.45<br>**6점**	19.91<br>**5점**	16.61<br>**4점**	14.03<br>**2점**	12.82<br>**2점**
구릉지<br>3.59	20.02<br>**6점**	19.74<br>**5점**	18.36<br>**5점**	17.05<br>**4점**	14.22<br>**2점**	12.01<br>**1점**	10.98<br>**1점**
급경사지<br>-	-	-	-	-	-	-	-

Explanation: The vertical flow of water is replaced by horizontal flow as the slope increases and the flow of water inside the soil. The slope is 2 to 4°, and shallow lowland and gorge are formed on the surface.

1. Conservation value of Wetland formed along the shoulder

Low	13 Experts(Median)	1	2	3	4	5	6	**7**	8	9	10	High
	1st Survey Result(individual)											
	Re-evaluation											

2. Conservation value of Open water formed along the shoulder

Low	13 Experts(Median)	1	2	3	4	**5**	**6**	7	8	9	10	High
	1st Survey Result(individual)											
	Re-evaluation											

3. Conservation value of Open water formed along the shoulder

Low	13 Experts(Median)	1	2	3	4	5	6	**7**	8	9	10	High
	1st Survey Result(individual)											
	Re-evaluation											

4. Conservation value of Grassland formed along the shoulder

Low	13 Experts(Median)	1	2	3	4	**5**	6	7	8	9	10	High
	1st Survey Result(individual)											
	Re-evaluation											

5. Conservation value of Agricultural land formed along the shoulder

Low	13 Experts(Median)	1	**2**	**3**	4	5	6	7	8	9	10	High
	1st Survey Result(individual)											
	Re-evaluation											

6. Conservation value of Developed land formed along the shoulder

Low	13 Experts(Median)	1	**2**	3	4	5	6	7	8	9	10	High
	1st Survey Result(individual)											
	Re-evaluation											

7. Conservation value of barrenland(rock) formed along the shoulder

Low	13 Experts(Median)	1	**2**	3	4	5	6	7	8	9	10	High
	1st Survey Result(individual)											
	Re-evaluation											

<Slope>

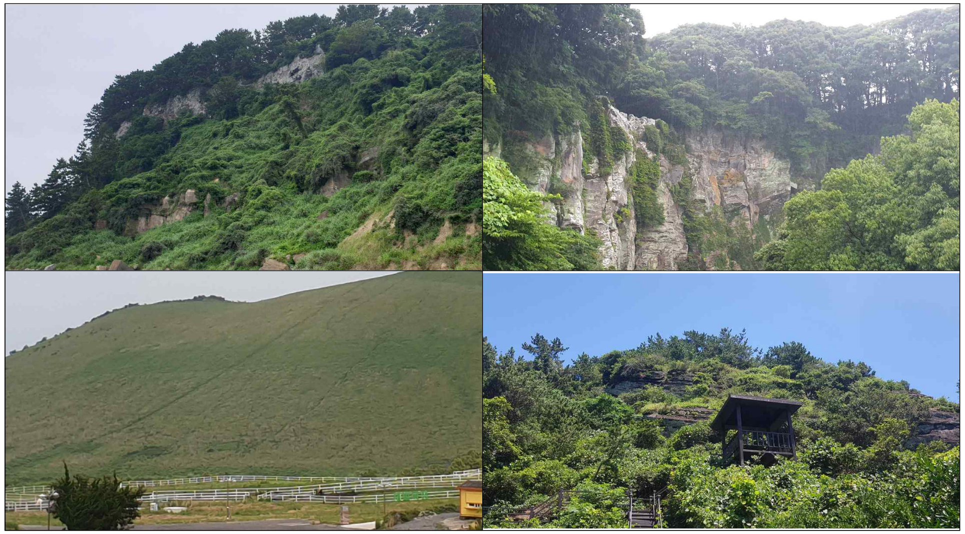

Explanation: Free surface with exposed basement rock generally indicates a slope of more than 45° and is usually more than 65°. Rock is dominant because of the extremely weak development of soil and vegetation due to steep slopes.

1. Conservation value of wetland formed along the slope

Low		1	2	3	4	5	6	7	8	9	10	HIgh
	Evaluation											

2. Conservation value of open water formed along the slope

Low		1	2	3	4	5	6	7	8	9	10	HIgh
	Evaluation											

3. Conservation value of forest formed along the slope

Low		1	2	3	4	5	6	7	8	9	10	HIgh
	Evaluation											

4. Conservation value of grassland formed along the slope

Low		1	2	3	4	5	6	7	8	9	10	HIgh
	Evaluation											

5. Conservation value of agricultural land formed along the slope

Low		1	2	3	4	5	6	7	8	9	10	HIgh
	Evaluation											

6. Conservation value of developed land formed along the slope

Low		1	2	3	4	5	6	7	8	9	10	HIgh
	Evaluation											

7. Conservation value of barrenland(rock) formed along the slope

Low		1	2	3	4	5	6	7	8	9	10	HIgh
	Evaluation											

<Flatland>

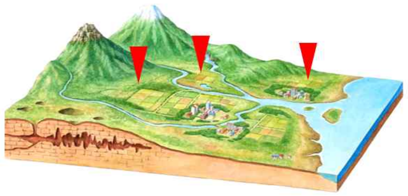

	습지 5.58	수역 5.50	산림지 5.12	초지 4.75	농업지역 3.96	시가화 건조지역 3.35	나지 (암반포함) 3.06
봉우리 5.19	28.96 10점	28.56 10점	26.56 9점	24.66 8점	20.57 6점	17.37 4점	15.88 3점
하천부지 4.62	25.74 9점	25.38 9점	23.61 8점	21.92 7점	18.28 5점	15.44 3점	14.11 2점
상부 4.19	23.38 7점	23.06 7점	21.45 6점	19.91 5점	16.61 4점	14.03 2점	12.82 2점
구릉지 3.59	20.02 6점	19.74 5점	18.36 5점	17.05 4점	14.22 2점	12.01 1점	10.98 1점
급경사지 -	-	-	-	-	-	-	-

Explanation: The midpoint of the upper slope and lower land where the outflow of material prevails and the inflow of material prevails at the same time. Erosion, slide, creep, and movement of material within the soil layer commonly occur. It shows the accumulation of substances from the upper soil topsoil topography surface and can observe distinct sediment layers as a result of long-term slope development.

1. Conservation value of wetland formed on a flat land

L	13 Experts(Median)	1	2	3	**4**	5	6	7	8	9	10	H
o	1st Survey Result(individual)											I
w	Re-evaluation											g h

2. Conservation value of open water formed on a flat land

L	13 Experts(Median)	1	2	3	4	5	**6**	7	8	9	10	H
o	1st Survey Result(individual)											I
w	Re-evaluation											g h

3. Conservation value of forest formed on a flat land

L	13 Experts(Median)	1	2	3	4	**5**	6	7	8	9	10	H
o	1st Survey Result(individual)											I
w	Re-evaluation											g h

4. Conservation value of grassland formed on a flat land

L	13 Experts(Median)	**1**	**2**	3	4	5	6	7	8	9	10	H
o	1st Survey Result(individual)											I
w	Re-evaluation											g h

5. Conservation value of agriclutural land formed on a flat land

L	13 Experts(Median)	1	**2**	3	4	5	6	7	8	9	10	H
o	1st Survey Result(individual)											I
w	Re-evaluation											g h

6. Conservation value of developed land formed on a flat land

L	13 Experts(Median)	**1**	2	3	4	5	6	7	8	9	10	H
o	1st Survey Result(individual)											I
w	Re-evaluation											g h

7. Conservation value of barren land(Rock) formed on a flat land

L	13 Experts(Median)	**1**	2	3	4	5	6	7	8	9	10	H
o	1st Survey Result(individual)											I
w	Re-evaluation											g h

<Channel>

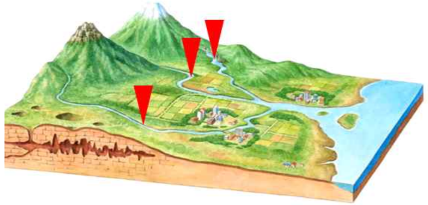

	습지<br>5.58	수역<br>5.50	산림지<br>5.12	초지<br>4.75	농업지역<br>3.96	시가화 건조지역<br>3.35	나지<br>3.06
봉우리<br>5.19	28.96<br>**10점**	28.56<br>**10점**	26.56<br>**9점**	24.66<br>**8점**	20.57<br>**6점**	17.37<br>**4점**	15.88<br>**3점**
하도<br>4.62	25.74<br>**9점**	25.38<br>**9점**	23.61<br>**8점**	21.92<br>**7점**	18.28<br>**5점**	15.44<br>**3점**	14.11<br>**2점**
상부<br>4.19	23.38<br>**7점**	23.06<br>**7점**	21.45<br>**6점**	19.91<br>**5점**	16.61<br>**4점**	14.03<br>**2점**	12.82<br>**2점**
완경사지<br>3.59	20.02<br>**6점**	19.74<br>**5점**	18.36<br>**5점**	17.05<br>**4점**	14.22<br>**2점**	12.01<br>**1점**	10.98<br>**1점**
급경사지<br>-	-	-	-	-	-	-	-

Explanation: Erosion and accumulation of matter by river flow occurs. The flow of water is always present in the case of higher-order streams, and the flow of water is intermittent in the case of lower-order rivers.

1. Conservation value of wetland formed along channel

L o w	13 Experts(Median)	1	2	3	4	5	6	7	**8**	9	10	H I g h
	1st Survey Result(individual)											
	Re-evaluation											

2. Conservation value of openwater formed along channel

L o w	13 Experts(Median)	1	2	3	4	5	6	7	**8**	9	10	H I g h
	1st Survey Result(individual)											
	Re-evaluation											

3. Conservation value of forest formed along channel

L o w	13 Experts(Median)	1	2	3	4	5	**6**	**7**	8	9	10	H I g h
	1st Survey Result(individual)											
	Re-evaluation											

4. Conservation value of grassland formed along channel

L o w	13 Experts(Median)	1	2	3	4	5	**6**	7	8	9	10	H I g h
	1st Survey Result(individual)											
	Re-evaluation											

5. Conservation value of agricultural land formed along channel

L o w	13 Experts(Median)	1	2	3	**4**	5	6	7	8	9	10	H I g h
	1st Survey Result(individual)											
	Re-evaluation											

6. Conservation value of developed land formed along channel

L o w	13 Experts(Median)	**1**	2	3	4	5	6	7	8	9	10	H I g h
	1st Survey Result(individual)											
	Re-evaluation											

7. Conservation value of barren land formed along channel

L o w	13 Experts(Median)	1	2	**3**	4	5	6	7	8	9	10	H I g h
	1st Survey Result(individual)											
	Re-evaluation											

<Appendix>

Summit	
Wetland	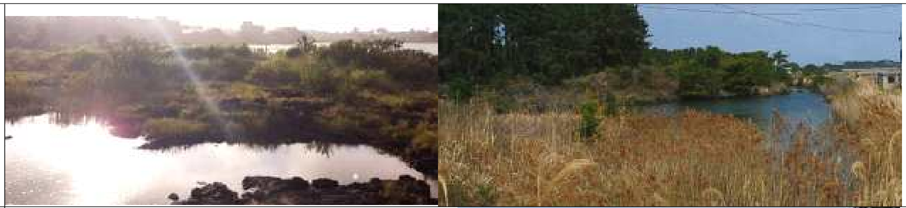
Open Water	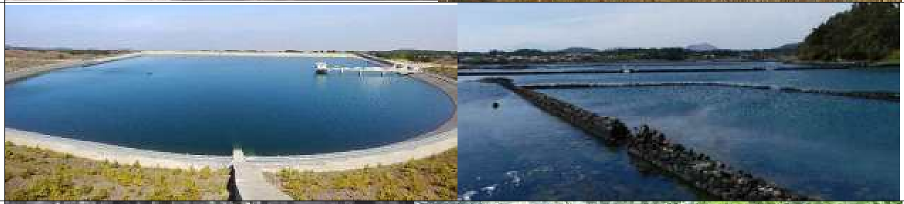
Forest	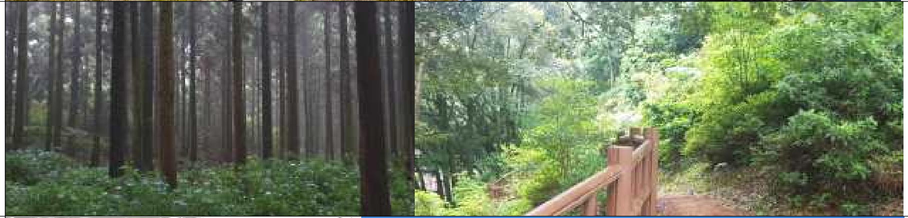
Grassland	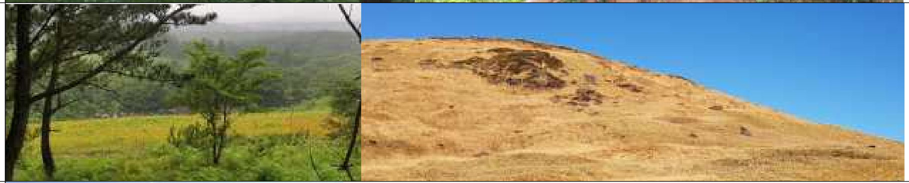
Agr° Land	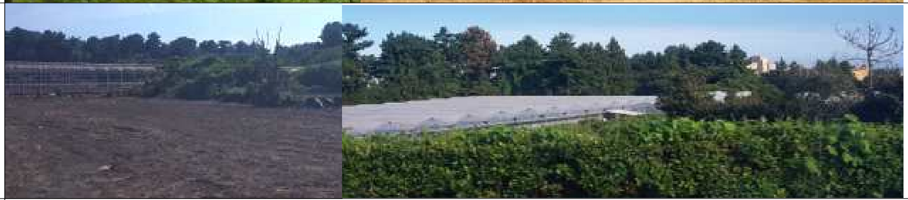
Developed land	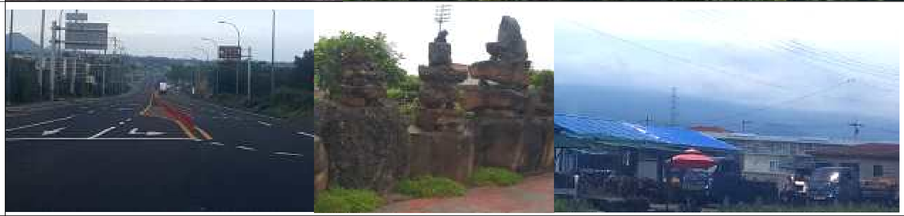
Barren Land	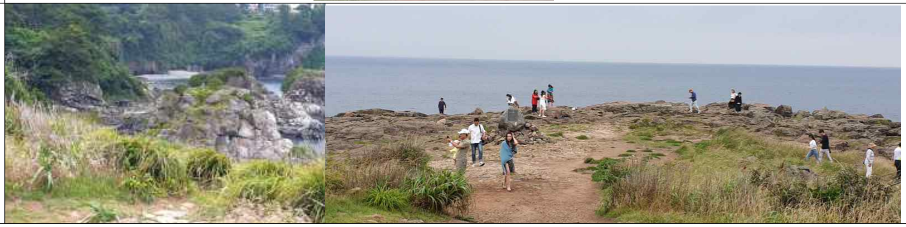

Shoulder	
Wetland	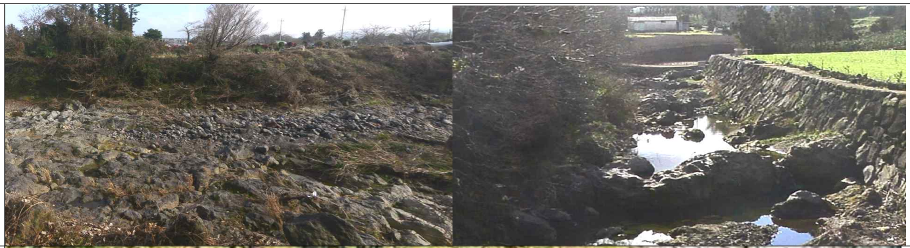
Open Water	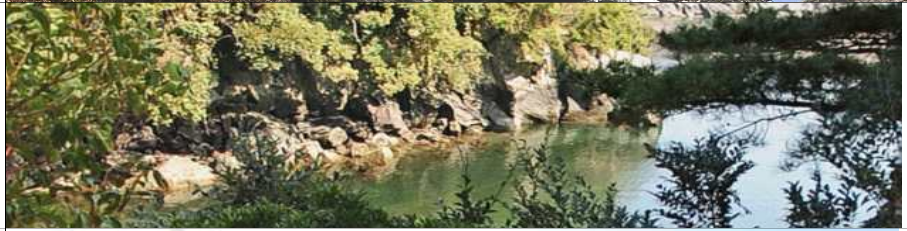
Forest	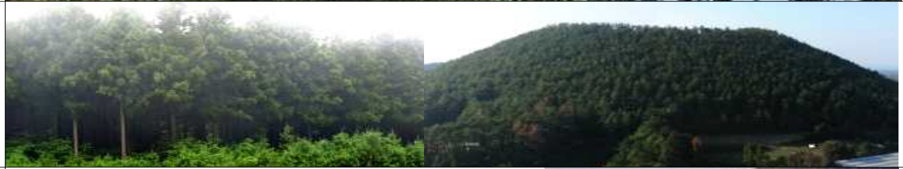
Grassland	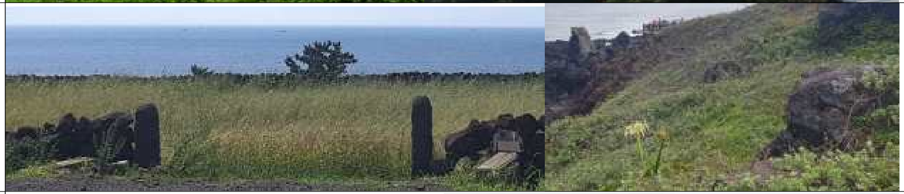
Agr° Land	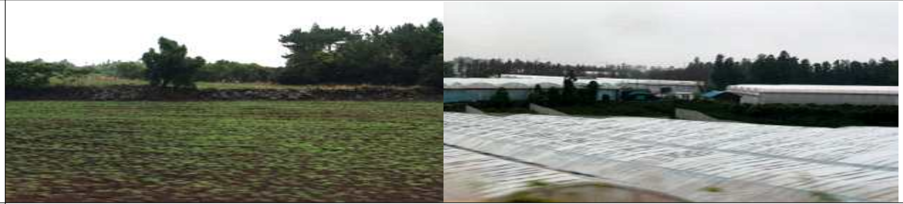
Developed land	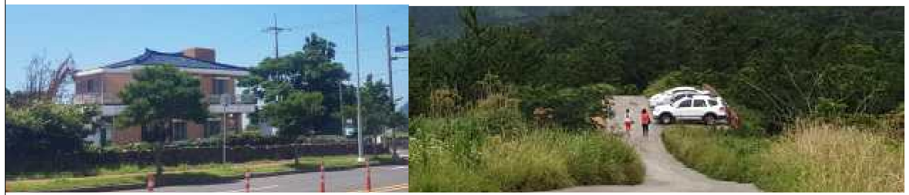
Barren Land	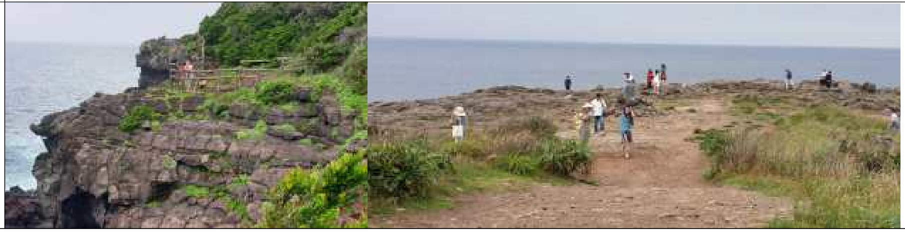

	Slope
Wetland	
Open Water	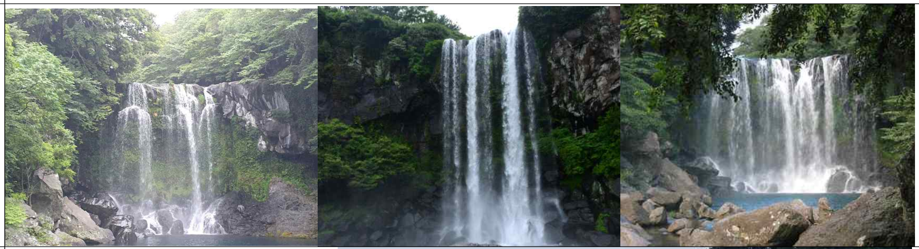
Forest	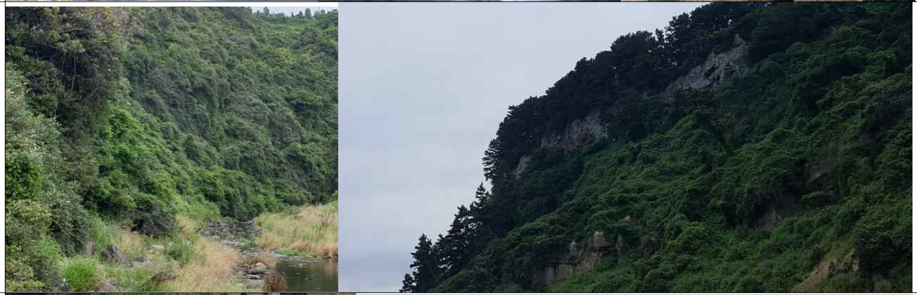
Grassland	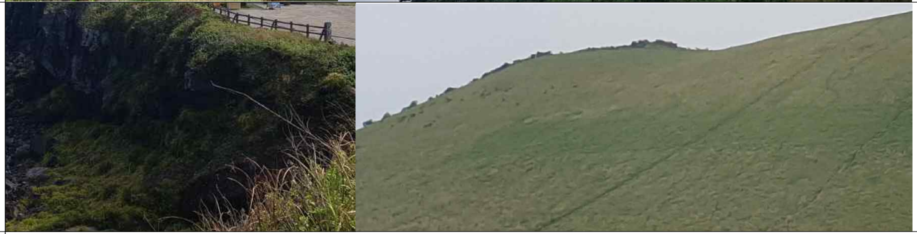
Agr° Land	
Developed land	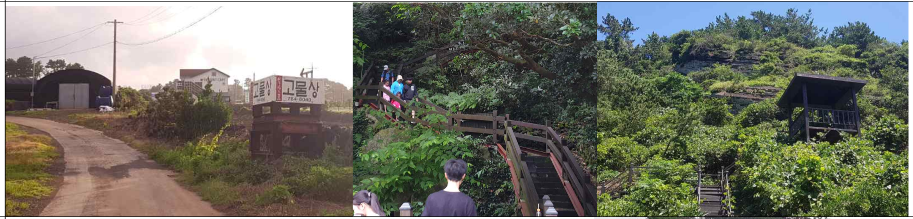
Barren Land	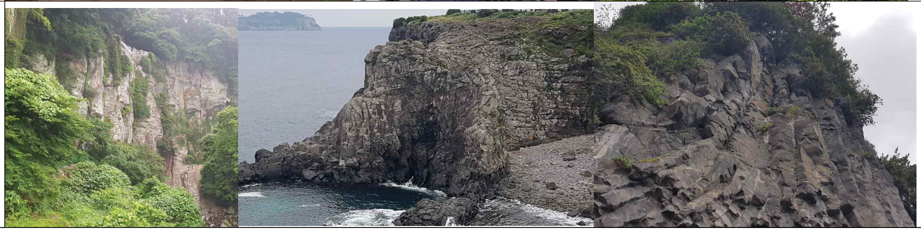

	Flat land
Wetland	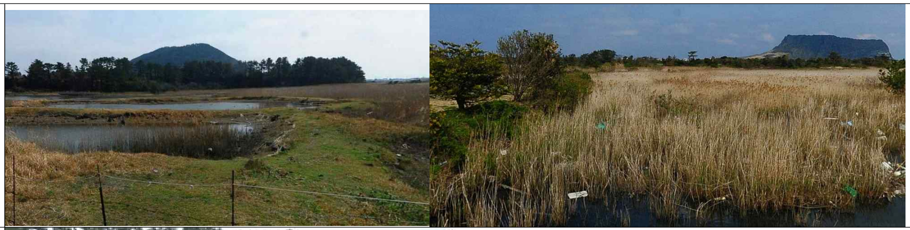
Open Water	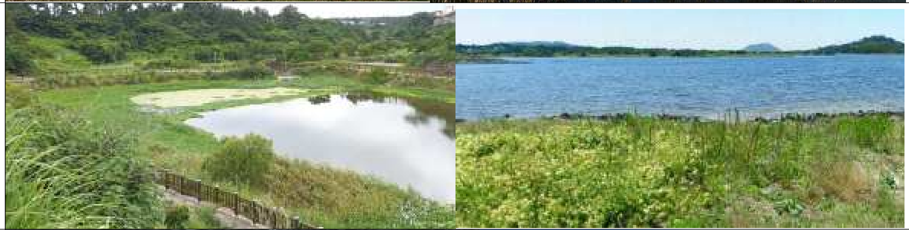
Forest	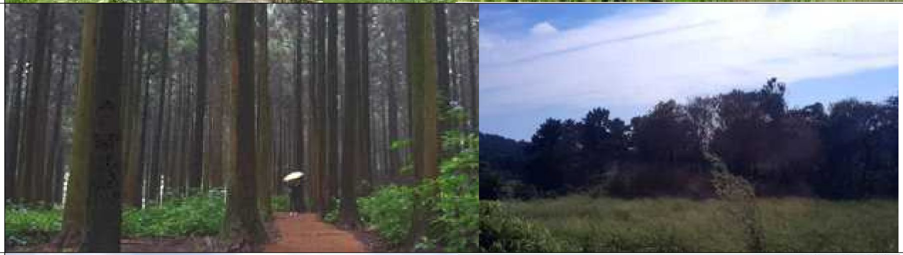
Grassland	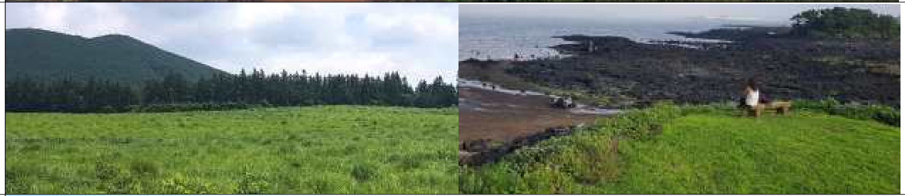
Agr° Land	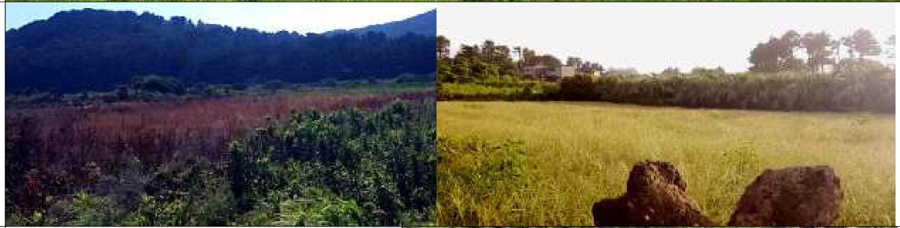
Developed land	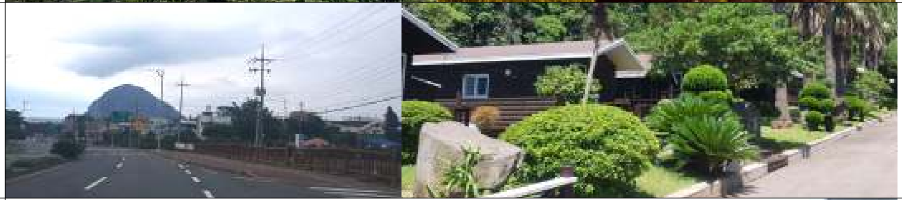
Barren Land	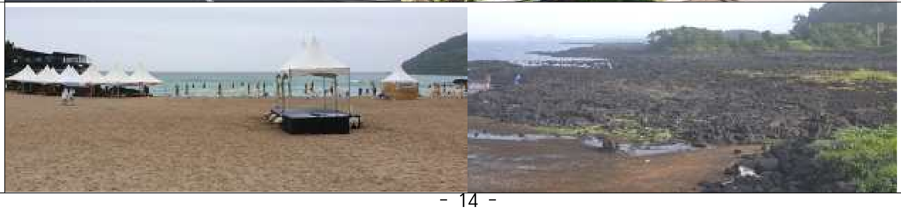

Channel		
Wetland	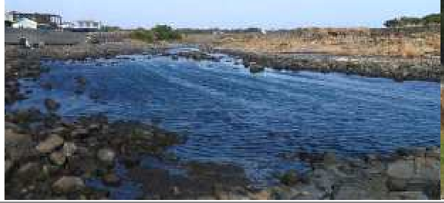	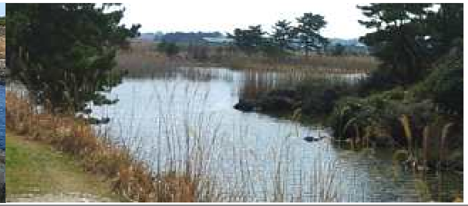
Open Water	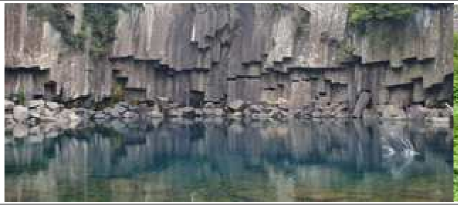	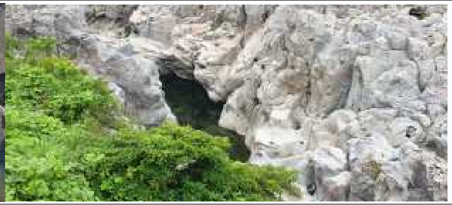
Forest	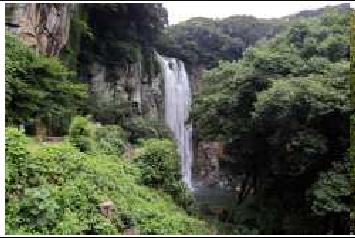	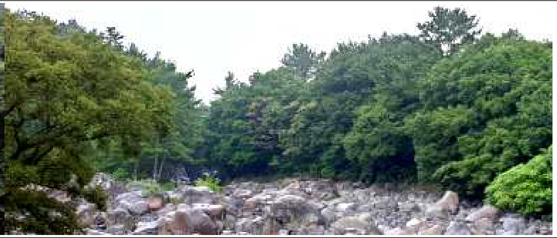
Grassland	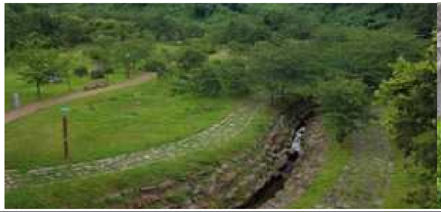	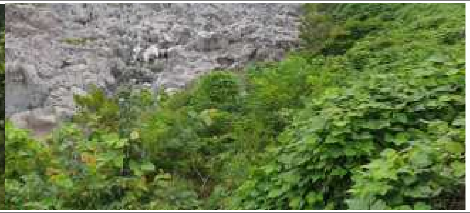
Agr° Land	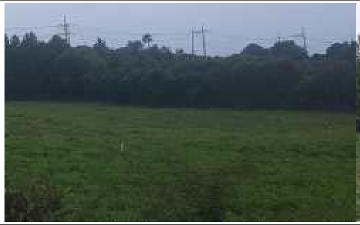	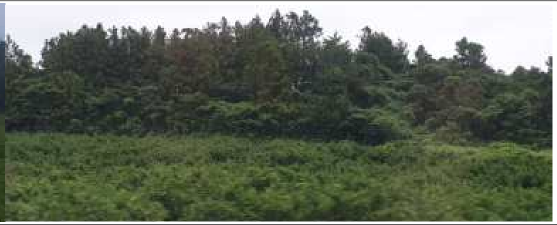
Developed land	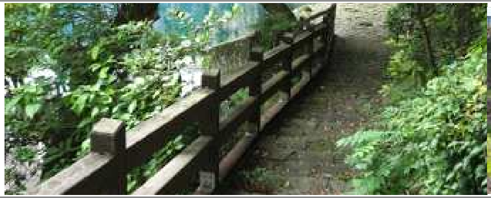	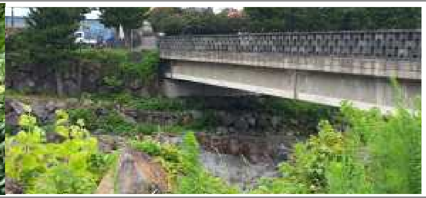
Barren Land	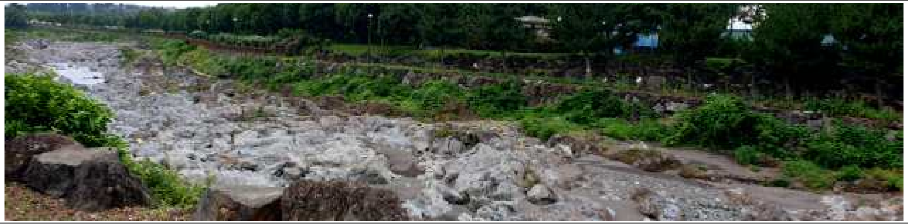	

Personal Information

Name	
Age	20s☐ 30s☐ 40s☐ 50s☐ 60s☐
Major	
Education	Bachelor☐ Master☐ Ph.D☐
Work Experience	( )years
Contact	Affiliation:
	Address:
	E-mail address:

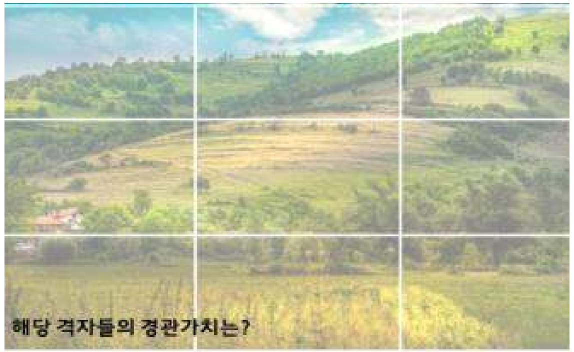

Thank you for your participation.

Please send your response to baysokjun@nie.re.kr within 7 days from the day you received this survey.
