# Supplementary material for: Development of landscape conservation value map of Jeju island, Korea for integrative landscape management and planning using conservation value of landscape typology
Source: PeerJ. 2021 Jun 1;9:e11449. doi: 10.7717/peerj.11449 (PMC8176906; doi:10.7717/peerj.11449)
Supplement: Supplemental Information 8 [file peerj-09-11449-s008.pdf]

1st Focus Group Interview Summary

Topic : Current limitations and potentials of landscape assessment approach

Anonymous (Geology Professor)

- For comprehensive landscape evaluation, topographical factors are not enough, hence various factors such as soil, lipids, and vegetation should be added to the evaluation for further research.

- The representativeness, scarcity, singularity, impossibility of reproducibility, academic and educational value, nature, diversity, scale, etc. that act on topographical landscape assessment items that are part of the national natural environment survey should be considered for evaluation.

- An assessment of the aesthetic value of the landscape should also be made, but since no basic research is currently in place, it is necessary to make a simple assessment through a combination of land coverings and topographical classification, and to include future additions to improve the indicators.

Anonymous (Landscape Professor)

- Landscapes consist of a medium of nature and scenery, so they are limited to evaluating landscapes by visual value alone and need to be carried out after an understanding of the intrinsic value of the landscape is made.

- Currently, the domestic environmental impact assessment project has numerous limitations as it is difficult to interpret the space objectively and cannot be expressed by drawing as the evaluation is limited to the visual value and analysis is conducted at every necessary moment.

- Cultural considerations play an important role, and the distribution status of tangible and intangible cultural assets should be considered, but it is too early to apply at the basic stage.

- Since basic research is insufficient to evaluate the intrinsic value and visual value of the landscape at once, a step-by-step approach is needed to evaluate the intrinsic value of the landscape followed by an evaluation of the visual value.

- At this point, it is expected that the use of land cover map and topographical classifications which briefly include cultural and ecological characteristics, will be appropriate from a macro perspective to identify the intrinsic value of the landscape. In addition, the DEM used for land cladding and terrain classification is highly utilized because the renewal cycle is stable.

Anonymous (Landscape expert)

- In Korea, the term landscape is mainly interpreted from an aesthetic point of view, but ecological and visual values must be considered for systematic evaluation of landscape.

- In order to create an objective map, subjectivity and randomness need to be excluded

as much as possible at the master level of mapping.

- In order to analyze the intrinsic value of the landscape, the interpretation of the ecological value of the landscape should be prioritized and the consideration of the visual value should be applied in the future.
- Landscape-related DBs have been established in various fields and agencies, but the data is not consistent to establish objective maps of the entire country.
- Landscape type is important before identifying the intrinsic value of the landscape, but it seems necessary to prepare a standard to derive it since the landscape type has not been identified at the moment.
- If the purpose of drawing is clearly set and the scale and user perspective are taken into account, the process of deriving the Landscape type will be easy.
