# Supplementary material for: Development of landscape conservation value map of Jeju island, Korea for integrative landscape management and planning using conservation value of landscape typology
Source: PeerJ. 2021 Jun 1;9:e11449. doi: 10.7717/peerj.11449 (PMC8176906; doi:10.7717/peerj.11449)
Supplement: Supplemental Information 9 [file peerj-09-11449-s009.pdf]

2nd Focus Group Interview Summary

Topic : What is a suitable way of defining intrinsic value of landscape

Anonymous (Landscape expert)

- Landscape scale approach is a basic step to grade the intrinsic value of the landscape and should be taken into account for its low precision while being comprehensive.
- Watershed has the characteristics of encompassing ecological and visual aspects, so the method of classifying the landscape as watershed may also be reasonable.
- The research site serves as a good pilot site because it has a lot of excellent natural resources compared to other regions, but it should be considered that locality can act as a variable in applying the same method to other regions in the future.
- While conducting an expert survey, clear and in-depth explanation of each landform type has to be provided as not all the landscape experts recognize differences between each landform types.

Anonymous (Landscape Professor)

- Differences in the hierarchy of interpretation (experimental approach, ecological approach, etc.) may occur if there are no clear evaluation criteria in the process of interpreting the intrinsic value of the landscape.
- In assessing the intrinsic value of landscape, experts need to present standards that reflect their own experience and knowledge and recommend using the 6 factors presented by the National Natural Environment Survey guidelines, which are familiar to most landscape experts.
- If the Landscape type is carefully reclassified, it can be linked to information such as biotope map, distribution, and accessibility in the future, and it is expected that a more realistic quantitative evaluation will be possible.
- Cultural Landscape and Natural Landscape have different meanings, and in order to identify the intrinsic value of the landscape, an approach that separates the two landscapes must be taken at the initial stage. Cultural landscape is an environment in which human behavior and environment work and form, and it seems that land cover map or land use map is suitable in the early stage, considering the application not only in the search site but also in other regions.
- Quantification of landscape value may be a great asset to policy establishment. Therefore, fundamental conceptualization of national landscape needs a careful consideration and has to be carried on step by step.

Anonymous (Landscape Professor)

- The usability of landscape value map should be carefully considered.
- The use of land cover, land use and landform map as factors will allow an objective approach.

- The method for defining intrinsic value of landscape should contain considerations for regionality.
- It is desperate that landscape value map to be prepared for dealing with diverse development activities.
- This research will potentially provide a landscape concept based ideas for screening wide range of development activities, and institutionalization of monitoring strategies.

Anonymous (Landscape Professor)

- The use of landscape value approach in relation to regulations should be considered.
- The landscape value approach may become a critical influence on survey quality and scale
- A clear purpose of this research should be defined whether it is going for a whole national territory or a site scale development projects.
- Landscape base map for national scale should be built prior to detailed approach since the larger scale approach allows a screening process.
- It is worth trying combining land cover types and landform types as long as the data stays updated constantly.
