# Supplementary material for: Development of landscape conservation value map of Jeju island, Korea for integrative landscape management and planning using conservation value of landscape typology
Source: PeerJ. 2021 Jun 1;9:e11449. doi: 10.7717/peerj.11449 (PMC8176906; doi:10.7717/peerj.11449)
Supplement: Supplemental Information 10 [file peerj-09-11449-s010.pdf]

3rd Focus Group Interview Summary

Topic : Final review on a 'Expert Survey form'

Anonymous (Landscape Professor)

- It is difficult to imagine specific landform types such as slope, mild slope, flat land. I suggest adding more sample images and rephrasing explanations using general terminologies.

- It may be helpful to reclassify land cover types with an actual land user perspectives. For instance, recreational zone included in developed land cover type may sound different to other survey participant.

- It would be better for survey participants to understand the characteristics of landform types if the explanations could deliver clear and direct messages along with sample images.

Anonymous (Landscape expert)

- The purpose of the survey needs to be clearly explained

- It would be better to rephrase explanation of landform types with more familiar terminologies rather than geological terminologies.

- It may be challenging task for participants to rate appropriate weighting rate since land cover and landform are both very important factors. Therefore, brief and clear statements are needed when asking for the weight.

Anonymous (Landscape Professor)

- The researcher need to be aware that sample images may not deliver a clear impression to the participants as land cover types in reality are similar to each other especially in Korea and so are landform types. Therefore, it is important to be careful to select most and realistic sample images. Yet current sample images are not enough. At least four images for each types are needed.

- The researcher need to clearly provide and explain standards (6 factors) for the evaluation.

- It may be a challenging task to have landscape experts to evaluate conservation value of land cover and landform types due to dense buildings and facilities with complicated and dynamic land use patterns within small territories. But it is worth trying since background map and current use of landscape resource data are limited.

- It will be more appropriate to have participants to measure land cover and landform type separately for the first survey then during the second survey participants need to rate the conservation value of landform and land cover type combinations.
